# The First Proteomic Study of *Nostoc* sp. PCC 7120 Exposed to Cyanotoxin BMAA under Nitrogen Starvation

**DOI:** 10.3390/toxins12050310

**Published:** 2020-05-09

**Authors:** Olga A. Koksharova, Ivan O. Butenko, Olga V. Pobeguts, Nina A. Safronova, Vadim M. Govorun

**Affiliations:** 1 Lomonosov Moscow State University, Belozersky Institute of Physical-Chemical Biology, Leninskie Gory, 1-40, 119992 Moscow, Russia; safronova.nina2007@mail.ru; 2 Institute of Molecular Genetics, Russian Academy of Sciences, Kurchatov Square, 2, 123182 Moscow, Russia; 3 Federal Research and Clinical Centre of Physical-Chemical Medicine, 119435 Moscow, Russia; ivan.butenko@gmail.com (I.O.B.); nikitishena@mail.ru (O.V.P.); vgovorun@yandex.ru (V.M.G.)

**Keywords:** nitrogen metabolism, heterocyst differentiation, amino acid metabolism, protein PII, NodM, glutamate metabolism, gltX, SecY, photosystems, oxidative stress

## Abstract

The oldest prokaryotic photoautotrophic organisms, cyanobacteria, produce many different metabolites. Among them is the water-soluble neurotoxic non-protein amino acid beta-N-methylamino-L-alanine (BMAA), whose biological functions in cyanobacterial metabolism are of fundamental scientific and practical interest. An early BMAA inhibitory effect on nitrogen fixation and heterocyst differentiation was shown in strains of diazotrophic cyanobacteria *Nostoc* sp. PCC 7120, *Nostoc* *punctiforme* PCC 73102 (ATCC 29133), and *Nostoc* sp. strain 8963 under conditions of nitrogen starvation. Herein, we present a comprehensive proteomic study of *Nostoc* (also called *Anabaena*) sp. PCC 7120 in the heterocyst formation stage affecting by BMAA treatment under nitrogen starvation conditions. BMAA disturbs proteins involved in nitrogen and carbon metabolic pathways, which are tightly co-regulated in cyanobacteria cells. The presented evidence shows that exogenous BMAA affects a key nitrogen regulatory protein, PII (GlnB), and some of its protein partners, as well as glutamyl-tRNA synthetase gltX and other proteins that are involved in protein synthesis, heterocyst differentiation, and nitrogen metabolism. By taking into account the important regulatory role of PII, it becomes clear that BMAA has a severe negative impact on the carbon and nitrogen metabolism of starving *Nostoc* sp. PCC 7120 cells. BMAA disturbs carbon fixation and the carbon dioxide concentrating mechanism, photosynthesis, and amino acid metabolism. Stress response proteins and DNA repair enzymes are upregulated in the presence of BMAA, clearly indicating severe intracellular stress. This is the first proteomic study of the effects of BMAA on diazotrophic starving cyanobacteria cells, allowing a deeper insight into the regulation of the intracellular metabolism of cyanobacteria by this non-protein amino acid.

## 1. Introduction

Cyanobacteria are the oldest microorganisms capable of oxygenic photosynthesis, and some of them fix atmospheric nitrogen. In many ecosystems, cyanobacteria are the primary producers of organic matter, and often they are symbionts of plants and animals [1]. Eutrophication of lakes and rivers leads to the development of cyanobacterial bloom and the production of many dangerous cyanotoxins [2]. Therefore, it is important to understand the biological functions of these cyanotoxins. The object of our research is a non-protein amino acid with neurotoxic action called beta-N-methylamino-L-alanine (BMAA). All known groups of cyanobacteria living both in an aquatic environment and on land, including symbiotic and free-living cyanobacteria [3] and diatoms [4], can synthesize this non-proteinogenic amino acid. BMAA is known to accumulate and biomagnify in plants, animals, and the human body and is associated with the development of human neurodegenerative diseases such as amyotrophic lateral sclerosis/parkinsonism-dementia complex [3,5,6,7]. Due to those undesirable consequences, BMAA actions have been studied mostly in mammalian models [7]. 

It is also known that BMAA significantly affects animal, plant, and cyanobacteria cells by suppressing or changing essential life functions such as motor neuron activity in animals, seedling development in plants, and photosynthesis and nitrogen fixation in cyanobacteria [7]. To date, the metabolic role of BMAA in cyanobacteria remains unclear. So far, information regarding BMAA synthesis and catabolism and its functional role in cyanobacterial cells is still very limited [7,8,9,10,11]. Only three investigations have demonstrated the biological impact of BMAA on nitrogen-fixing filamentous cyanobacterium *Nostoc* (also called *Anabaena*) sp. PCC 7120 [12,13,14]. *Nostoc* sp. PCC 7120 is known as a model nitrogen-fixing organism with a complete genome sequence and well-developed genetic techniques [15]. 

It is important to underline that the functional state and development of *Nostoc* cells depend on the growth conditions. When combined nitrogen (nitrate or ammonium) is available, cyanobacterial filaments contain only vegetative cells. During diazotrophic growth in nitrogen-limited conditions, this cyanobacterium uses specialized cells called heterocysts that protect nitrogenase, a nitrogen fixation enzyme, from oxygen. Heterocysts provide neighboring vegetative cells with nitrogenous compounds and, in turn, receive reduced carbon compounds from vegetative cells, which serve as source of energy and reducing agents [16]. Between these two main physiological states (nitrogen replete and diazotrophic growth conditions), there is an important special transition period when cyanobacteria form heterocysts from vegetative cells. This process of cell differentiation is unique for cyanobacteria and is triggered by intracellular signals of nitrogen deficiency (nitrogen starvation). This developmental process is genetically regulated and depends on the activity of several regulatory proteins [16]. This cell differentiation includes many significant intracellular events in the vegetative cells before they become heterocysts. Among them are genomic rearrangement, photosystem II reorganization, special polysaccharide capsule formation, and many others [16]. The biological effect of BMAA on cyanobacterial cell function was demonstrated in these three physiological conditions in early studies, in which different experimental methods were applied [12,13,14]. 

It was found that the addition of BMAA inhibited nitrogenase activity in mature heterocysts of diazotrophic *Nostoc* (*Anabaena*) sp. PCC 7120 [12,13]. The use of real-time PCR allowed the discovery of inhibited expression of a nitrogenase-specific gene, *nifH,* by BMAA in this cyanobacterium [13]. Furthermore, under nitrogen starvation, at the start of heterocyst differentiation, adding BMAA inhibited this process. As a result, heterocysts did not form and the cyanobacteria died of starvation. It was shown that during nitrogen deprivation, BMAA downregulates transcription of the key heterocyst-specific genes *hetR* and *hepA* in *Nostoc* [13]. An unusual opposite regulatory effect of BMAA on diazotrophic cyanobacteria was found in nitrogen-replete conditions [14]. Normally, when combined nitrogen is available, heterocyst formation is blocked and cyanobacterial filaments contain only vegetative cells. However, micromolar amounts of BMAA induced heterocyst-specific gene expression and formation of heterocyst-like nonfunctional cells under repressive conditions in nitrogen-replete *Nostoc* [14]. It could be that adding BMAA to nitrogen-sufficient vegetative cells induces some kind of intracellular signal of nitrogen deficiency [14]. This, in turn, leads to the expression of genes that are usually silent in the presence of combined nitrogen. Therefore, in these two studies it was demonstrated that exogenous BMAA influences nitrogen metabolism and gene expression of filamentous nitrogen-fixing cyanobacteria. The cyanotoxin disturbs their normal development. Specifically, under nitrogen starvation, cyanotoxin suppresses [13] heterocyst formation, while under nitrogen-replete conditions, it induces the formation of heterocyst-like cells and expression of heterocyst-specific genes [14]. The reasons for such regulation are still unknown. 

Proteomic analysis can complement and expand the previously described functional changes that occur in cyanobacterial cells under BMAA action [12,13,14]. So far only two -omics studies have been performed to investigate the impact of BMAA on eukaryotic cells: a proteomic analysis of zebrafish exposed to sublethal doses of BMAA [17] and a metabolic study of human neuroblastoma cells under BMAA treatment [18]. We performed, for the first time, comparative proteomic studies of the impact of BMAA on cyanobacteria cell protein profiles in all of the physiological conditions mentioned above. 

The aim of this proteomic study was to examine the biological effects of exogenous BMAA on nitrogen-starving cyanobacterium *Nostoc* (*Anabaena*) sp. PCC 7120 (further referred to as *Nostoc*) during heterocyst formation. 

## 2. Results and Discussion 

### 2.1. Proteins Regulated by BMAA under Nitrogen Starvation Conditions

Altogether, 1567 proteins of nitrogen-starving *Nostoc* were identified (Supplementary Table S1). Among them, 103 proteins belonging to different functional categories were selected for further analysis and discussion based on the statistical significance of the observed differences between BMAA-treated and control samples (Table 1 and Table 2, Supplementary Table S2). 

The selected proteins are presented in Table 2 and Supplementary Table S2. Each table contains the following information: the name of the identified protein, corresponding gene number, metabolic pathway or function (with an exception for hypothetical proteins), fold change between BMAA-treated and control samples, *p*-value. Overall, 32 proteins were shown to be downregulated more than two-fold in BMAA-exposed cells; 7 were observed only in the control samples and were absent in the BMAA-treated samples (Table 1 and Table 2, Supplementary Table S2). Sixteen proteins were upregulated more than two-fold in BMAA-treated cells. Proteins, effected by this cyanotoxin, are involved in different metabolic pathways in cyanobacteria cells, among them are nitrogen and carbon metabolism, heterocyst development, photosynthesis, amino acid synthesis, protein synthesis and stress response, signaling, DNA repair and metabolism, transcription, and secondary metabolite biosynthesis (Table 1 and Table 2). A large fraction of the identified proteins (35 proteins) were classified as “hypothetical” proteins (Table 1, Supplementary Table S2). Modern bioinformatics tools permit to find clues to possible functions of the identified proteins. For example, ALCOdbCyano (http://alcodb.jp/cyano/) is a bioinformatics database that can help find information about genes, which coexpress with genes encoding the identified hypothetical proteins. This database contains coexpression data for three model cyanobacteria, including *Nostoc*. Thus, the gene coexpression data for the identified hypothetical proteins in the proteome of nitrogen-starving *Nostoc* under BMAA treatment were found according to ALCOdbCyano and are shown in Supplementary Table S3. 

A more detailed discussion of the selected proteins specified by their functional category (Table 2) can be found below.

### 2.2. Nitrogen Status Sensing, Transport, and Assimilation 

**Figure 1:**
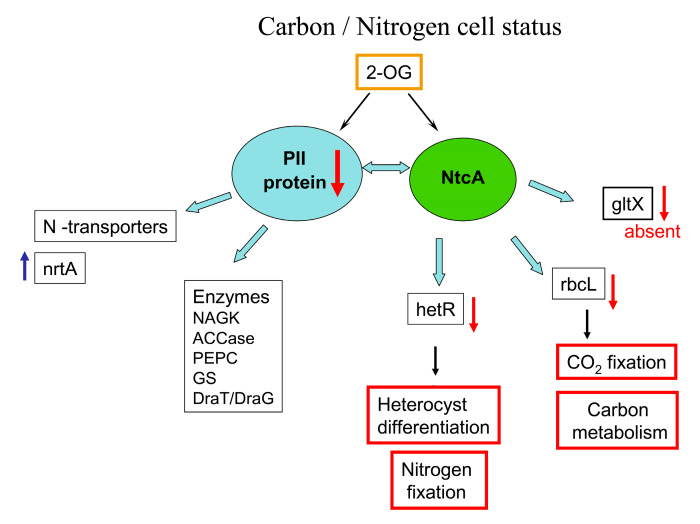
Protein PII as a key regulator in carbon/nitrogen metabolism in *Nostoc* cells. The main targets of protein PII action are presented on the scheme. Light-blue arrows represent interactions between protein PII and its main protein partners [19]. Red arrows stand for downregulation of proteins (↓) and a blue arrow stands for upregulation of a protein (↑). Differently regulated by BMAA proteins and genes were identified in this study and in our previous work [13].

Cyanobacteria are photoautotrophic organisms that are able to maintain cellular homeostasis by sensing and regulating their intracellular carbon/nitrogen (C/N) balance. They possess a finely regulated signal transduction network, which involves 2 oxoglutarate (2-OG)—a metabolic intermediate and an important signaling molecule [19]. 2-OG participates in glutamate and glutamine synthesis and plays a key role in cellular C/N balance sensing. Nitrogen from different nitrogen sources (including nitrate and/or N_2_ for diazotrophic strains) is converted into ammonium, which is then incorporated into the carbon skeleton of 2-OG for the biosynthesis of various biomolecules in cyanobacterial cells. The accumulation of 2-OG in cells signals nitrogen starvation and triggers the formation of nitrogen-fixing heterocysts in diazotrophic cyanobacterial strains. There are two well-studied receptors of 2-OG in the nitrogen-starvation signaling pathways: trimeric protein PII and transcription factor NtcA. 2-OG modifies the activity of PII signaling protein and NtcA transcription factor (Figure 1) [19]. 2-OG and other effectors (the ATP (adenosine triphosphate) or ADP (adenosine diphosphate) molecules enable the PII T-loop to adopt various conformations to interact with diverse protein partners [19]. 2-OG leads as well to a conformational change of NtcA—a key transcription factor in the nitrogen and carbon metabolism of cyanobacteria, and by this enhances NtcA’s DNA-binding ability.

We found that BMAA affects the regulation of key nitrogen regulatory protein PII (GlnB) (*all2319*) and several other proteins involved in nitrate transport and heterocyst differentiation and functioning (Table 2). PII protein was downregulated in BMAA-treated samples. First identified in 1969 as a component of the glutamine synthetase regulatory apparatus, sensor-transducer protein PII plays a key role in the control of nitrogen metabolism in prokaryotic cells [20]. It is known to pass and transform signals of abundance or deficiency of carbon, nitrogen, and energy that lead to changes in the activity of enzymes, channels, regulatory proteins, and gene expression (Figure 1) [19,20,21]. PII protein can modify the catalytic activity of enzymes involved in nitrogen metabolism [19]. 

**Figure 2:**
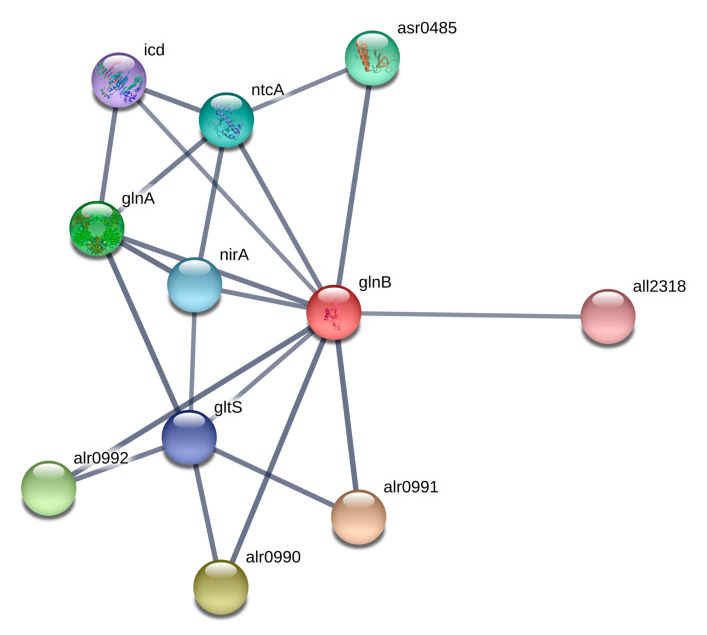
Protein network of nitrogen regulatory protein PII (GlnB) and its protein partners according to STRING (https://string-db.org), where alr0990, alr0991, alr0992 are ammonium transporters; gltS is Glutamate synthase; glnA is Glutamine synthetase; nirA ferredoxin is nitrite reductase; ntcA is Global nitrogen regulator and transcriptional activator of genes subject to nitrogen control; icd is isocitrate dehydrogenase; asr0485 is PII interaction protein X; all2318 is RNA-binding protein TAB2.

The PII signal-transduction protein is involved in the regulation of various nitrogen- and carbon-anabolic processes through binding to various targets [19,21,22,23]. PII plays a central role in the control of various nitrogen-related enzymes, transcription factors and transporters (Figure 1; Figure 2) [23]. Remarkably, addition of BMAA also changes the amount of nitrate transport nitrate-binding protein nrtA (*alr0608*) (Table 2), which is a partner of the PII protein [19,23] (Figure 2). NrtA is upshifted in *Nostoc* cells under BMAA treatment in the absence of nitrate in the growth media. Considering the downregulation of PII protein (Table 2) and its negative regulation of nitrate transporters [23], the observed upregulation of NrtA protein by BMAA seems logical. Note that the transcription of the *nirA* gene, encoding nitrite reductase, is also upregulated under 48 h BMAA treatment in nitrogen-starving cells of *Nostoc* [13].

Note that PII protein was also indicated in association with the RNA-binding protein TAB2, which is encoded by *all2318* (Figure 2). The TAB2 protein participates in the regulatory pathway of light-controlled photosystem protein synthesis in plants [24]. This protein is required for translation of *psaB* mRNA in chloroplasts [25]. In our study, we found that the *psaB* protein (*alr5155*) was downshifted 3.88-fold in BMAA-treated cells during nitrogen stepdown (Table 2). We can hypothesize that the decrease in the amount of PII protein can have an effect on the regulation of its protein partners, in particular TAB2 (*all2318*). Experimental verification of the TAB2 regulatory effect on Photosystem I (PSI) protein synthesis is an interesting new subject for future investigation.

In this study, we identified several interesting hypothetical proteins that are impacted by BMAA in *Nostoc* cells. Using the ALCOdbCyano database (http://alcodb.jp/cyano/) we found that the *all2319* gene, encoding downregulated protein PII (Table 2), is on the top of the same coexpressed gene list as the *all2375* and *all2080* genes, which encode the identified downregulated hypothetical proteins (http://alcodb.jp/cyano/PCC7120/all2319/list) (Supplementary Tables S2 and S3). The coexpression of these three genes and similar regulation of their protein products in nitrogen-starving *Nostoc* cells in the presence of BMAA allow us to suggest that the PII regulatory protein may have two more uncharacterized functional partners (corresponding genes *all2375* and *all2080*). These two proteins could be used as new targets for future experimental studies.

Summarizing the above, we can note that BMAA influences the regulation of the key regulatory protein PII and some of its functional protein partners. PII is downshifted and nitrate transport nitrate-binding protein (NrtA) is upshifted in BMAA-treated cells of *Nostoc* in the absence of nitrate (under nitrogen starvation). Taking into account that the global transcriptional regulator NtcA is an important partner of PII (Figure 1 and Figure 2) [26], it can be proposed that the one possible reason for the BMAA inhibitory effect on *hetR* and *hepA* gene expression [13] may be its negative effect on PII protein and subsequent changes in NtcA protein activity. In turn, this protein is the master regulator of genetic responses to the C/N balance at the transcriptional level in cyanobacteria [27,28]. NtcA is a global regulatory transcription factor that directly regulates the expression of multiple genes required for nitrogen and carbon assimilation, and genes involved in a number of other metabolic pathways, such as DNA metabolism, transcription and translation, and central metabolism [28]. In our study, 17 proteins were found that are encoded by genes, which are under the control of NtcA (*alr0608*, *all2319*, *all1454*, *alr0599*, *alr4380*, *alr0140*, *all4662*, *all5263*, *alr1524*, *alr5275*, *alr4566*, *alr4505*, *all1411*, *asl4547*, *alr2889*, *asr3294*, *all4662*) (Table 2, Supplementary Table S2).

### 2.3. Heterocyst Differentiation and Nitrogen Fixation

*Nostoc* is a filamentous cyanobacterium that can fix atmospheric nitrogen and plays a key role in the global nitrogen turnover [29]. Nitrogenase enzyme converts atmospheric nitrogen into ammonium in specialized anti-oxygen protected cells (heterocysts). These specialized cells form when sources of combined nitrogen are scarce. Heterocyst formation and nitrogenase synthesis are genetically and metabolically controlled under nitrogen stepdown [28]. In our proteomic study, we found that under BMAA treatment, the DNA binding protein Abp2 (*all1939*) [30] was downshifted (Table 2). This protein is very important for expression of *hepC* and *hepA* genes and subsequent heterocyst maturation in *Nostoc* (*Anabaena*) [30]. As we demonstrated earlier, Abp2 mutation leads to full inactivation of *hepC* and *hepA* gene expression and prevents heterocyst maturation and aerobic nitrogen fixation. As we showed using thin-layer chromatography of lipid extracts and transmission electron microscopy, Abp2^–^ mutant did not form heterocyst envelope glycolipids and can not fix nitrogen in an oxygen-containing milieu [30]. Moreover, BMAA downregulates a product of the *nifD* gene (nitrogenase molybdenum-iron protein, subunit alpha) (Table 2). Previously, we demonstrated by real-time PCR that, during nitrogen starvation, BMAA inhibits expression of another structural gene, *nifH* that encodes the other functional component of the nitrogenase enzyme, dinitrogenase reductase, in *Nostoc* [13]. Both structural genes of nitrogenase, *nifD* and *nifH,* are downregulated in the presence of BMAA during nitrogen stepdown. 

It should also be taken into account that BMAA downregulates two hypothetical proteins encoded by genes *alr2440* and *alr3297* identified in this study (Supplementary Table S2) that are co-expressed with two heterocyst differentiation related genes, *hetR* (*alr2339*) and *patN* (*alr4812*), respectively (Supplementary Table S3). It can be proposed that these two downshifted hypothetical proteins *(alr2440* and *alr3297*) may also participate in the process of heterocyst formation. These two genes could be selected as new targets for the future experimental investigation of their biological functions.

The presented proteomic results are in good agreement with our previously published gene expression data [13] and confirm the inhibitory effect of BMAA on heterocyst development and nitrogen fixation. This negative effect could be due to the downregulation of PII protein as shown experimentally. This protein interacts with other proteins, including transcriptional factor NtcA, (Figure 1 and Figure 2, Supplementary Figure S1), therefore changes in PII amount can lead to changes in nitrogen regulation and carbon metabolism of *Nostoc* under the action of BMAA. This subject requires further detailed experimental elucidation. 

### 2.4. Inorganic carbon Uptake and Assimilation

In cyanobacteria, carbon acquisition takes place mainly via CO_2_ fixation reactions. Ribulose-1,5-bisphosphate carboxylase/oxygenase (RubisCO) is the central CO_2_ fixation enzyme; it uses CO_2_ (carboxylase activity) and O_2_ (oxygenase activity) as substrates. Efficient carbon fixation through RubisCO in phototrophic cyanobacteria relies on the ability to concentrate inorganic carbon (Ci) near the RubisCO active site. Cyanobacteria possess CO_2_-concentrating mechanisms (CCMs) to facilitate carbon fixation by the RubisCO enzyme. The signal molecule 2-oxoglutarate α-ketoglutarate (2-OG) is used by cyanobacteria as a status reporter of the C/N balance, such that under low nitrogen conditions (high 2-OG levels), the CCM would be tuned down, which is in agreement with the diminished rate of CO_2_ fixation under nitrogen-deprived conditions (Figure 1) [19]. 

In our study, two annotated proteins involved in CO_2_ fixation were downregulated by adding BMAA to nitrogen-starving *Nostoc* (Table 1 and Table 2): a large subunit of ribulose bisophosphate carboxylase (rbcL; EC: 4.1.1.39, gene *alr1524*) (Supplementary Figure S2) and ccmM protein (*all0865*), involved in the carbon dioxide concentration mechanism [31,32]. According to STRING, ccmM protein interacts with ribulose bisphosphate carboxylase large chain (rbcL) (Supplementary Figure S3). Therefore, CO_2_ fixation in *Nostoc* in nitrogen-starving conditions was downshifted due to the lower amount of the two essential proteins. 

In the *Nostoc* proteome we found three hypothetical proteins (encoded by genes *all1361*, *all2080*, *alr2440*; Supplementary Table S2) that may be involved in common cellular processes with the carbon dioxide concentration mechanism proteins CcmM and CcmK (Supplementary Table S3). These three hypothetical proteins were also downregulated under the effect of BMAA in nitrogen-depleted cells of *Nostoc* (Supplementary Table S2). 

Six enzymes involved in carbon assimilation pathways were identified as downshifted proteins. Among them are phosphoglycerate kinase (pgk), fructose-1,6-bisphosphatase I, 6-phosphogluconate dehydrogenase, and phosphoglucomutase (Table 2, Supplementary Figures S4 and S5). 

### 2.5. Secondary Metabolite Synthesis

Two enzymes involved in thiamine metabolism were absent in BMAA-treated samples of nitrogen-starving cyanobacteria cells (Table 2, Supplementary Figure S6): 1-deoxy-xylulose 5-phosphate synthase (EC: 2.2.1.7) and thiazole synthase (thiG, EC: 2.8.1.10), which participate in the same pathway as thiamine biosynthesis. Thiamine pyrophosphate (TPP), the active form of thiamine, is a cofactor for enzymes involved in the central metabolic pathways: in the pentose phosphate pathway, the tricarboxylic acid cycle, and the synthesis of amino acids. Thiamine also participates in protection against biotic and abiotic stresses [33]. 

In addition, 1-deoxy-xylulose 5-phosphate synthase (EC: 2.2.1.7) is a key enzyme in terpenoid backbone biosynthesis [34]. This thiamin diphosphate (ThDP)-dependent enzyme catalyzes the decarboxylative condensation of pyruvate and d-glyceraldehyde 3-phosphate (d-GAP) to form 1-deoxy-d-xylulose 5-phosphate. This metabolite is involved in three separate essential pathways for central bacterial metabolism: ThDP synthesis, pyridoxal phosphate (PLP) synthesis, and the methylerythritol phosphate (MEP) pathway for isoprenoid synthesis [35].

Two other enzymes (4-hydroxy-3-methylbut-2-en-1-yl diphosphate synthase and geranylgeranyl hydrogenase) were downshifted two-fold (Table 2). They also participate in terpenoid and chlorophyll biosynthesis and metabolism. Chlorophyll a is the main pigment of cyanobacterial photosynthesis; therefore, an imbalance in the photosynthetic apparatus of *Nostoc* can be expected. Indeed, the photosynthetic machinery is strongly affected by BMAA (details are reported in Section 2.6). 

### 2.6. Photosynthesis

Cyanobacteria are the first organisms that were able to use oxygenic photosynthesis to convert carbon dioxide into different organic chemicals [29]. As mentioned, cyanobacteria sense and regulate the intracellular carbon/nitrogen balance. However, BMAA treatment obviously changes this regulation. Several proteins involved in photosynthesis were downregulated or upregulated in the presence of BMAA (Table 2). Among the downregulated proteins were the main proteins of photosystem I, some proteins of pigment complexes, and plastocyanin (petE) (Table 2, Supplementary Figure S7). PsaA (photosystem I P700 chlorophyll a apoprotein A1) was downregulated almost three-fold and PsaB (photosystem I P700 chlorophyll a apoprotein A2) was downregulated almost four-fold. These two proteins are encoded by two genes that are coexpressed, *alr5154* and *alr5155* (http://alcodb.jp/cyano/PCC7120/alr5154/list). At the same time, one protein of photosystem II (13 kDa protein, psbW) and protein petC (cytochrome b6-f complex iron–sulfur subunit) were upregulated (Table 2, Supplementary Figure S7). In this regard, it can be mentioned that PII protein is covalently modified in conditions of imbalanced photosynthetic electron transfer in cyanobacterium *Synechococcus* sp. PCC 7942 when photosystem II predominates over photosystem I [36]. It was suggested that the photosynthetic electron transport chain may regulate the nitrogen assimilation pathway in cyanobacteria cells by means of posttranslational modification of PII protein [36]. In our proteomic study, we also saw significant changes in the amount of proteins representing the key parts of the photosynthetic apparatus of *Nostoc* as well as downregulation of PII protein (Table 2).

BMAA also affects chlorophyll metabolism in cyanobacterial cells. Three enzymes, delta-aminolevulinic acid dehydratase (EC: 4.2.1.24), glutamyl-tRNA synthetase (EC: 6.1.1.17), and geranylgeranyl hydrogenase (EC: 1.3.1.111), are involved in chlorophyll metabolism. They are strongly downregulated by BMAA (Table 2, Supplementary Figure S8).

We can conclude that adding BMAA leads to very strongly disordered cyanobacterial photosynthesis and nitrogen fixation. As mentioned, changes during the differentiation of vegetative cells to heterocysts are complex and well regulated. The oxygen-producing photosystem II disappears but photosystem I remains intact in heterocysts to protect oxygen-sensitive nitrogenase from inactivation. This means that light can be harvested and used to generate chemical energy (ATP), which is required for nitrogen fixation in heterocysts. The heterocysts also become dependent on neighboring vegetative cells for the reduction of equivalents (electrons) that are imported in the form of sucrose [37]. However, in the presence of BMAA the main proteins of photosystem I are strongly downregulated in cyanobacteria cells. 

BMAA downregulation of nitrogen fixation, carbon assimilation, and photosynthesis results in cyanobacterial cell starvation for nitrogen, carbon, and energy.

### 2.7. Amino Acid Metabolism

Considering significant changes induced by BMAA in the amount of proteins involved in nitrogen metabolism, CO_2_ fixation, and photosynthesis, it can be assumed that these changes will affect the synthesis and metabolism of amino acids as well. Indeed, BMAA induces multiple changes in the regulation of eight enzymes that participate in amino acid synthesis and metabolism. Five proteins are downregulated and three proteins are upregulated. Adding BMAA to nitrogen-starving cyanobacterial cells changes the regulation of enzymes involved in valine, leucine, isoleucine, and arginine biosynthesis (Table 2). Additionally, BMAA disturbs enzymes, which participate in several amino acid metabolism pathways. 

Specifically, BMAA affects enzymes involved in alanine, aspartate, and glutamate metabolism, as was also detected in human neuroblastoma cells by metabolic profiling [18]. Two enzymes, glucosamine-fructose-6-phosphate aminotransferase (NodM) and succinate-semialdehyde dehydrogenase, are upregulated in the presence of BMAA in nitrogen-starving cells of *Nostoc* (Table 2, Supplementary Figure S9). Both enzymes participate in glutamate metabolism. The enzyme NodM (*alr3464*) participates in glutamate metabolism and aminosugar metabolism. The two substrates of this enzyme are L-glutamine and D-fructose 6-phosphate, whereas its two products are L-glutamate and D-glucosamine 6-phosphate. NodM protein participates in the GlnA and GlnB (PII) protein network (Supplementary Figure S10). NodM is upshifted in *Nostoc* under BMAA treatment in all three growth conditions discussed above. This enzyme (EC: 2.6.1.16) is upregulated after BMAA treatment during nitrogen starvation (Table 2), in the nitrogen-replete medium [38] and in diazotrophic conditions when *Nostoc* already possesses mature heterocysts (before BMAA treatment) during continued growth in a nitrogen-free medium [39]. We can hypothesize that somehow BMAA, possibly acting as a glutamate analog (for review, see [7,11]), is able to change the regulation of the glutamate metabolism enzymes.

The second upshifted enzyme, succinate-semialdehyde (*all3556*), has three substrates, succinate semialdehyde, Nicotinamide adenine dinucleotide (NAD+), and H_2_O, whereas its three products are succinate, NADH, and H+. This enzyme belongs to the family of oxidoreductases, specifically those acting on the aldehyde or oxo group of donors with NAD+ or NADP+ as an acceptor. This enzyme participates in glutamate and butyrate metabolism. In bacteria, the enzyme is also involved in γ-aminobutyric acid (GABA) degradation, but it can be recruited to facilitate other functions, such as converting succinate-semialdehyde formed during fission of the pyridine ring to succinic acid for entry into the Krebs cycle [40].

BMAA has a notable effect on glycine, serine, and threonine metabolism (Supplementary Figure S11). Three enzymes involved in that pathway are downregulated and one is upregulated. Threonine dehydratase is encoded by *alr4232* and is absent in BMAA-treated cells (Table 2, Supplementary Figure S11). This pyridoxal-phosphate protein catalyzes the deamination of threonine to 2-ketobutyrate and ammonia. It can play a biosynthetic or biodegradative role. In the former role, the enzyme supplies 2-ketobutyrate required for isoleucine biosynthesis, while in the latter threonine dehydratase is only involved in the breakdown of threonine to supply energy. Another enzyme, phosphoserine aminotransferase (EC: 2.6.1.52), encoded by *all1683*, is downshifted in the presence of BMAA. This enzyme is also a pyridoxal-phosphate protein, and it catalyzes two reversible reactions. In one, O-phospho-L-serine and 2-oxoglutarate are metabolized into 3-phosphonooxypyruvate + L-glutamate. In the other reaction, the enzyme converts 4-phosphonooxy-L-threonine and 2-oxoglutarate into (3R)-3-hydroxy-2-oxo-4-phosphonooxybutanoate and L-glutamate. It should be noted that 2-oxoglutarate participates in both reactions. Its regulatory and signal functions were discussed above.

The homoserine dehydrogenase thrA (*all 4120*) is upregulated in BMAA-treated cells of *Nostoc* under nitrogen starvation. This enzyme conducts the reduction of aspartate beta-semialdehyde into homoserine. The aspartate metabolic pathway is involved in the storage of asparagine and synthesis of aspartate-family amino acids [41]. Homoserine is an intermediate in isoleucine, methionine, and threonine biosynthesis.

It is worth noting that arginine is the amino acid with the highest nitrogen content and is frequently a key element of nitrogen storage compounds in photosynthetic organisms. However, one enzyme involved in arginine biosynthesis is slightly downshifted in BMAA-treated cells, urease UreC (*alr3670*). Ureases catalyze the hydrolysis of urea into ammonia and carbon dioxide. 

BMAA also disturbs cysteine and methionine metabolism in cyanobacteria cells under nitrogen starvation (Table 2, Supplementary Figure S10). Two enzymes that participate in this pathway, phosphoserine aminotransferase (EC: 2.6.1.52) and cysteine synthase A (EC: 2.5.1.47), are downregulated (Table 2). These enzymes work coherently. Phosphoserine aminotransferase was discussed before. Cysteine synthase is a key enzyme in cysteine production. This amino acid is the main precursor of glutathione biosynthesis. Glutathione is a reducing tripeptide (cysteine, glutamic acid, and glycine) that protects proteins from denaturation that occurs due to oxidation of thiol groups in proteins under different stresses [42,43].

Summarizing, we can note that adding BMAA to nitrogen-starving cells of *Nostoc* leads to many changes in the regulation of enzymes that participate in amino acid metabolism, including pathways where glutamate and 2-oxoglutarate are the key players.

### 2.8. ABC Transporters

ATP binding cassette (ABC) transporters are energized by ATP and can transport complex organic molecules against concentration gradients. They can be either importers or exporters in bacterial cells and transport a variety of compounds across cell membranes. ABC exporters are involved in the formation of additional cell envelope layers and in the transmission of developmental signals [44,45,46]. ABC importers take part in obtaining various nutrients and metal ions. Some ABC transporters do not have transport functions. These proteins participate in repair of DNA and in mRNA translation [47]. We found that under BMAA treatment two transporters were downregulated (Table 1 and Table 2). One was the product of the *alr2535* gene. It was suggested that *alr2535* belongs to the gene cluster that encodes transporters that mediate the uptake of mainly hydrophobic amino acids. This protein can be involved in the uptake of Gly, Pro, and Glu in *Nostoc* [46].

Another transport protein, preprotein translocase subunit SecY (*all4197*), was found only in the control samples and was absent in the BMAA-treated samples (Table 2). The Sec translocase pathway is a main bacterial pathway of protein translocation across the cytoplasmic membrane from the cytosol. The core of the *Escherichia coli* translocase includes SecY, SecE, and SecG [48]. As deduced from the The UniProt Knowledgebase (UniProtKB) (https://www.uniprot.org/uniprot/A0A2K8WLB4), the cyanobacterial Sec translocase has an analogous composition of the core (SecY, SecE, and SecG). We need to take into account that cyanobacteria possess not only a cell envelope membrane, but also thylakoid membranes (an endomembrane system). SecY protein was localized in both cytoplasmic and thylakoid membranes of the *Synechococcus* PCC7942 [49]. Therefore, exportation from the cytosol could occur into the periplasm or the thylakoid lumen. Moreover, SecY protein binds to several ribosomal proteins (Table 3, Section 2.9) and participates in translation. Therefore, it can be assumed that the lack of SecY transporter in BMAA-treated cyanobacteria cells can lead to serious disturbances in many aspects of cellular metabolism. It could be a fascinating topic for future investigations.

One ABC transporter is upshifted in BMAA-treated samples (Table 2). This protein is encoded by the *alr0140* gene and is a peptide/nickel transport system substrate-binding protein. It could be involved in the quorum sensing (QS) pathway (https://www.genome.jp/kegg-bin/show_pathway?ana02024+alr0140). The biological function of this transporter is unknown. The proposed participation of this protein in QS is rather interesting and requires experimental verification.

To summarize, we can say that the ABC transporters of *Nostoc*, directly energized by ATP, play different significant roles in the complex lifestyle of this cyanobacterium [47]. It was found that cyanotoxin BMAA strongly downregulated two ABC transporters in nitrogen-starving *Nostoc*, which could lead to disturbances in amino acid and peptide transportation.

### 2.9. Ribosomal Proteins and Translation

As we reviewed previously, the biological impact of BMAA on living organisms [7], its effect on eukaryotic and prokaryotic cells, is pleiotropic and can involve different mechanisms. For example, it disturbs protein synthesis. In human cells, BMAA is mistakenly incorporated into proteins instead of L-serine [50]. Recently it was observed that BMAA is a substrate for human alanyl-tRNA synthetase (AlaRS) and can form BMAA-tRNAAla by escaping from the intrinsic AlaRS proofreading [51]. Moreover, for the first time, by using AlaRS from *Nostoc* (*Anabaena*) sp. PCC 7120, it was reported that cyanobacterial AlaRS also activates BMAA [51]. It is not yet known how inhibition of aaRS activity or mistranslation might contribute to the disturbance of cyanobacterial metabolism and heterocyst development.

We found, in the present proteomic study, that two proteins involved in translation were remarkably downregulated in the presence of BMAA (Table 2). Glutamyl-tRNA synthetase (gltX, *all3205*) was absent in starving cyanobacterial cells after the addition of BMAA. This important enzyme is involved in protein and pyrrole derivative biosynthesis. It may also be involved in bacterial persistence [52]. Moreover, in cyanobacteria *Synechococcus* sp. PCC 7942, transcription of *gltX* is activated under nitrogen sufficiency (in the presence of ammonium or nitrate) but not in nitrogen-free medium [53]. NtcA is needed for full *gltX* expression, but it is not required for basal transcription of this gene, which is consistent with the essential role of *gltX* in protein and pyrrole derivative biosynthesis. It was found that gltX, which is involved in the incorporation of glutamate (a product of the GS-GOGAT cycle) in protein synthesis, is under NtcA control, thus reflecting the involvement of NtcA in coordinated regulation of essentially every aspect of nitrogen metabolism. NtcA controls the expression of many genes, including genes encoding transporters (amt1, nrtA–D, nrtP, ureA–E), signal transduction regulators (glnB(PII), ntcA), and different enzymes [28,54]. Some genes from the latter group play key roles in the GS-GOGAT cycle, such as *glnA* encoding GS itself, which is involved in glutamine synthesis, and *icd* gene encoding isocitrate dehydrogenase, which is required for the synthesis of 2-oxoglutarate (a substrate of GOGAT). The metabolism of *Nostoc* is under global regulation that is determined by the availability and balance of carbon and nitrogen [55].

Another protein, LepA (*all2508*), a strongly conserved protein, is one of the noncanonical GTPases implicated in translation and important for bacterial growth and functional protein biosynthesis [56], but its functions are not fully understood [57]. LepA is an elongation factor that is suspected to improve the fidelity of translation by recognizing ribosomes with mistranslocated tRNA and consequently inducing back-translocation [58]. This protein was absent in BMAA-treated samples of nitrogen-starving *Nostoc* (Table 2).

These two proteins, gltX and LepA, are interconnected with several ribosome proteins (Table 3). Moreover, SecY protein, or preprotein translocase subunit SecY (*all4197*), which was also missing under BMAA treatment (Table 2), binds to several ribosomal proteins as well (Table 3). Note that all three proteins (gltX, LepA, and SecY) interact with the L6 protein, which participates in the ribosome function that is involved in the codon recognition process [59]. In this connection, it is interesting that L6 has been shown to be part of the aminoacyl-tRNA binding site and associated with the EF-G and EF-Tu binding sites [60]. Consequently, the absence of the three proteins that are important for the translation process (glutamyl-tRNA synthetase, LepA as an elongation factor, and Sec translocon forming protein SecY) in BMAA-treated samples definitely induces disorder in protein synthesis.

Moreover, we found that BMAA also affects several ribosomal proteins in *Nostoc*. Three ribosomal proteins (S7, S10, L12) were downregulated and two proteins (S6, L24) were upregulated in the BMAA-treated samples (Table 2). Remarkably, S10 protein, a component of the small ribosome subunit, was downregulated 14.6-fold. Together with other ribosomal proteins, L10 produces a tight complex and forms an extended ribosome stalk, which performs the important function of recruiting GTP-binding translation factors [61]. 

S10, gltX, and LepA proteins connect to the ribosomal protein L2 (Table 3). L2 is a conserved r-protein that occurs in the vicinity of the peptidyltransferase center in the 50S subunit and is essential for translational activity of the ribosome. Moreover, L2 is involved in tRNA binding in the A (aminoacyl) and P (peptidyl) sites [61].

Therefore, strong downregulation of these three proteins has a strong impact on protein synthesis in cyanobacterial cells.

In addition to the main function in the protein synthesis machinery, many ribosomal proteins have other functions, acting either as individual regulatory proteins or in complexes with other proteins [62]. The S10 protein was the first bacterial ribosomal protein found to participate in transcription regulation [63,64]. In bacterial cells, transcription and translation are tightly coupled; they are physically associated [65,66] and the key role in determining the transcription rate is played by the ribosome that follows RNA polymerase and translates the mRNA during its synthesis [66]. Information about additional functions of ribosomal proteins in cyanobacteria is scanty. Recently, it was reported that the cyanobacterial ribosome-associated protein LrtA of *Synechocystis* sp. PCC 6803 participates in post-stress survival of this cyanobacterium [67]. Notably, a recent review [68] presents the current findings in cyanobacteria, demonstrating the existence of versatile riboregulatory mechanisms that are involved in the control of the C/N balance. It is a relatively new and perspective research area of cyanobacterial investigation.

Summarizing, we can see from this proteomic study that BMAA may be able to disrupt the integrity of protein synthesis through multiple mechanisms.

### 2.10. Signaling, Stress Response Proteins, Proteases, and Chaperones

Taking into account the previously discussed effects of BMAA on various metabolic processes, especially photosynthesis, nitrogen fixation, and CO_2_ fixation, oxidative stress occurring in cyanobacteria cells due to intercellular metabolic and energetic imbalances can be expected [69].

Indeed, adding BMAA to nitrogen-starving cells of *Nostoc* upregulated three chaperones (Table 2) and four main enzymes involved in oxidative stress response. Among them are peroxiredoxin (*alr4641*), superoxide dismutase (*all0070*), glutathione reductase (*all4968*), and thioredoxin reductase (*all0737*). Note that superoxide dismutase, which dismutates O_2_^–^ to hydrogen peroxide, was identified only in BMAA-treated samples and not in control samples. These data may indicate the presence of severe intracellular oxidative stress resulting from the BMAA addition. The observed stress response of enzyme upregulation is in agreement with the data obtained in studies of eukaryotic cells demonstrating oxidative stress induction due to the addition of BMAA [11,70].

At the same time, in our proteomic study, we found that BMAA significantly reduced the amount of peroxiredoxin of the AhpC/TSA family (Table 2). This may indicate that antioxidant enzymes of *Nostoc* act independently, as this takes place in other Gram-negative bacteria [71]. It is worth noting that the *alr4404* gene encoding the AhpC/TSA protein is coexpressed with the *alr2440* gene that encodes downregulated hypothetical protein (Supplementary Tables S2 and S3). This hypothetical protein is very similar to saccharopine dehydrogenase-like oxidoreductase (96% identity, according to BLAST). In plants, saccharopine dehydrogenase-like oxidoreductase together with lysine-ketoglutarate reductase form one bifunctional enzyme [72]. Therefore, this hypothetical cyanobacterial protein could be an interesting new target for future experimental functional studies.

Under intracellular stress conditions, protease activity is increased to repair damaged proteins and protein complexes [73,74,75]. In our study, we found that the ATP-dependent zinc metalloprotease FtsH (*all4936*) was upshifted in the presence of BMAA (Table 2). This enzyme is a cytoplasmic membrane protein that has N-terminally located transmembrane segments and a main cytosolic region consisting of Zn^2+^-metalloprotease and AAA-ATPase domains [73]. FtsH controls the quality of integral membrane proteins, degrades short-lived proteins, and maintains cellular regulation at the level of protein stability. This protease degrades some misassembled membrane proteins. FtsH has a special ability to dislocate membrane protein substrates out of the membrane [74]. It degrades a few membrane proteins that have not been assembled into complexes, such as SecY, F0 ATPase subunit a, and others (https://www.uniprot.org/uniprot/P0AAI3). Note that in our proteomic research we found that SecY was absent in the BMAA-treated *Nostoc* cells (Table 2). Moreover, some cyanobacterial FtsH metalloproteases are directly involved in degrading damaged D1 proteins of PSII [75]. An increase in the amount of FtsH protease may indicate the presence of many misassembled membrane proteins in starving *Nostoc* cells under BMAA treatment. 

Therefore, stress response mechanisms are strongly upregulated in nitrogen-starving *Nostoc* after BMAA addition. 

### 2.11. DNA Metabolism and Transcription

As a result of the strong oxidative stress induced by BMAA, one can expect DNA damage to occur [76]. Indeed, BMAA affects all aspects of DNA metabolism. The cyanotoxin changes nucleotide metabolism, DNA repair, and DNA transcription (Table 2).

Nucleotide metabolism is central to all living organisms due to its essential role in DNA building and energy transfer. BMAA disturbs four enzymes involved in nucleotide metabolism; two are upregulated and two are downregulated (Table 2). This effect may be due to a general disturbance in carbon and nitrogen metabolism caused by the BMAA addition. Phosphoribosylglycinamide formyltransferase-2 (EC: 2.1.2.2) is upregulated two-fold in the presence of BMAA. It can be proposed that a possible effect of this amino acid on the glutamate–glutamine balance in cyanobacterial cells can also affect this enzyme regulation. The nitrogen molecules of the purine ring are derived from the amide group of glutamine and the amino groups of glycine and aspartate. Phosphoribosylglycinamide formyltransferase-2 performs one of the purine synthesis steps involving ATP and glutamine. The detailed mechanism of BMAA regulation should be explored in future experiments.

Moreover, adding BMAA to nitrogen-starving cells of *Nostoc* upregulates enzymes involved in DNA repair. The RecA protein and two subunits of DNA gyrase are upshifted three- and two-fold, respectively (Table 2). It can be suggested that the oxidative stress induced by BMAA may lead to DNA damage and, therefore, may induce DNA cell repair activity. 

Three main proteins involved in DNA transcription are also affected by BMAA treatment. RNA polymerase sigma factor RpoD (gene *sigA*) is downshifted 2.5-fold (Table 2) under stressful BMAA treatment. Previously, it was shown [77] that sigA mRNA, encoding the primary sigma in *Synechocystis* sp. strain PCC 6803, accumulated under standard growth conditions, while in stress conditions such as high salinity or heat sigA mRNA content decreased rapidly. 

Two subunits of DNA-directed RNA polymerase, rpoB and rpoC1, are upregulated (Table 2) in *Nostoc* in the presence of BMAA. Such induction of rpoB and rpoC1 may be explained by strong stress conditions induced by this cyanotoxin. It was also found that the expression level of *rpoB* and *rpoC1* genes is induced by various stresses in plants [78].

The presented results demonstrate a strong biological effect of BMAA on nitrogen-starving cyanobacterial cells. These experimental results may be helpful and can be taken into account when studying the effects of BMAA on cells of other organisms.

### 2.12. Hypothetical Proteins

In our proteomic study, we found 35 hypothetical proteins; almost half were upshifted and half were down-shifted (Table 1, Supplementary Table S2). It is possible to find some useful information about several hypothetical proteins (Supplementary Table S3) using ALCOdbCyano (http://alcodb.jp/cyano/), which displays coexpressed gene lists. Moreover, several hypothetical proteins are on the same list as proteins identified in the present proteomic study (Supplementary Table S3, marked in green).

Among upshifted hypothetical proteins there are several (alr1346, all1411, alr4505, alr0652) whose genes are coexpressed with chaperone genes (Supplementary Table S3). They may be involved in the stress response resulting from the presence of BMAA.

Among the downshifted hypothetical proteins are several interesting candidates for further study. Downregulated hypothetical proteins encoded by *all2375* and *all2080* are coexpressed together and with the *all2319* gene, which encodes PII protein downregulated in this study (Table 2, Supplementary Table S3). Downregulated hypothetical proteins encoded by *alr2440* and *alr3297* are on the same lists as the heterocyst differentiation related *hetR* (*alr2339*) and *patN* (*alr4812*) genes, respectively. Three downregulated hypothetical proteins encoded by *alr2440*, *all1361*, and *all2080* are found on the same lists as the genes encoding the carbon dioxide concentration mechanism proteins CcmK (*all0867*), CcmM (*all0865*), and CcmK (*alr0318*) (Supplementary Table S3). Notice that downregulated CcmM (*all0865*) protein is identified in our study (Table 2). 

Information about identified proteins reveals that some upshifted hypothetical proteins could be involved in stress response, while some downshifted hypothetical proteins could participate in nitrogen metabolism, heterocyst formation, and carbon fixation. The identified hypothetical proteins could be interesting as new targets for insertion mutagenesis and transcriptional analysis in upcoming studies.

## 3. Conclusions 

The results stated in this paper demonstrate the remarkable pleiotropic regulatory effect of β-N-methylamino-L-alanine (BMAA) on cyanobacterium *Nostoc* sp. PCC 7120 proteome under nitrogen starvation conditions. New proteomic data support and extend our previously published experimental results [13] by showing that BMAA disturbs proteins involved in nitrogen metabolism and in heterocyst differentiation and heterocyst functioning. It was shown, for the first time, that this cyanotoxin downregulates the key nitrogen regulatory protein PII as well as some of its protein partners, which are involved in nitrogen and carbon metabolism. Thus, these data bring us closer to the understanding the mechanisms underlying the action of BMAA on cellular differentiation of diazotrophic cyanobacteria. We propose that the main primary targets of the BMAA action are, apparently, metabolic processes involving 2-oxyglutarate, glutamate, regulatory protein PII. That is, acting as a glutamate analog, BMAA disturbs these metabolic processes, and probably NtcA transcriptional regulator.

**Figure 3:**
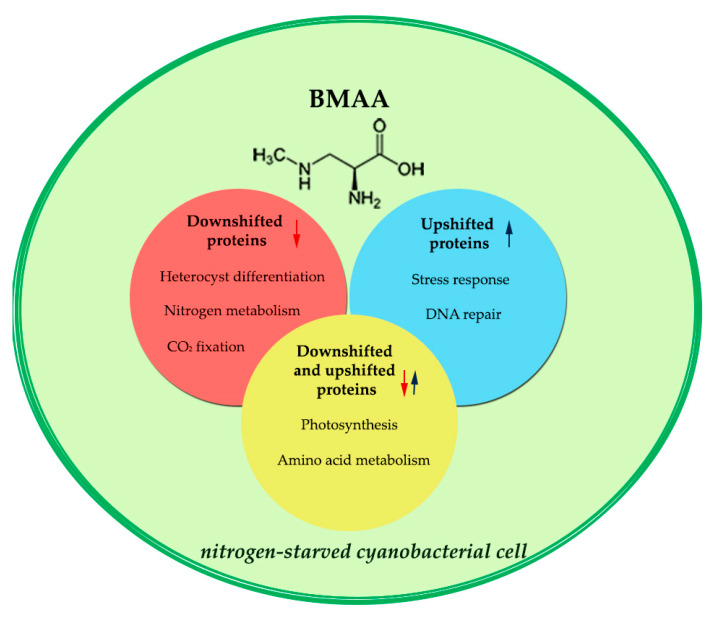
BMAA effect on proteins of the main processes and metabolic pathways in cyanobacterium *Nostoc* during nitrogen starvation. In the red circle area are stated categories of downregulated proteins involved in several processes: nitrogen metabolism, carbon fixation, heterocyst differentiation. In the blue circle area are placed categories of upregulated stress response and DNA repair proteins. Proteins of photosynthesis and amino acid metabolism are regulated differently (some proteins are upshifted, others are downshifted).

Moreover, we have discovered that BMAA suppresses proteins involved in all major metabolic pathways in cyanobacterial cells, causing strong intracellular stress. BMAA treatment leads to a disturbance of photosynthesis, carbon fixation and the carbon dioxide concentration mechanism, amino acid metabolism, protein synthesis, cell signaling. The existence of such internal stress is evidenced by the upregulation of cell protective mechanisms, which activate, in turn, oxidative stress defense proteins, DNA repair enzymes, chaperone proteins, proteases, and several signal proteins (Figure 3). In this study, it was shown for the first time for cyanobacteria that BMAA induces upregulation of the DNA repair enzyme, RecA, which is involved in inducible SOS response system of DNA repair and, therefore, can lead to bacterial apoptosis [79]. Indeed, cyanobacteria, experiencing starvation and strong stress under the BMAA action, undergo cell death and lysis [12,13].

Thereby a question arises: what is BMAA for cyanobacteria, if it kills them? Could it play a role in their density regulation in a natural microalgae community? Perhaps, BMAA can play a role of an infochemical molecule and it could be used, for example, by non-diazotrophic unicellular cyanobacteria and diatoms as an allelopathic tool in their competition for the nitrogen resource. It is known that unicellular non-dizotrophic strains of *Microcystis* and *Synechocystis* synthesize BMAA in the course of nitrogen starvation, while the addition of ammonium or nitrate abolishes this synthesis [8]. It is also known that natural competitors of cyanobacteria—diatoms—synthesize BMAA in large amounts [4,80,81]. As a result of the action of BMAA, a part of the dinitrogen fixators population undergoes lysis and dissolved organic nutrients necessary for the algae community are released. Thus, the obtained data are important for further fundamental investigations devoted to clarifying the regulatory roles of the neurotoxic amino acid BMAA in cyanobacterial metabolic networks and its possible ecological impact on interrelationships in algae communities. Future studies can more clearly pinpoint the regulatory role of BMAA in cyanobacterial cells. The information presented in this paper could be useful for upcoming experimental studies with the application of other tools, such as insertion mutagenesis, enzyme activity measurement, biosensor-based quantification, and transcriptional analysis. 

## 4. Materials and Methods

### 4.1. Cyanobacterial Strain and Cultivation Conditions

Filamentous nitrogen-fixing cyanobacterium *Nostoc* sp. PCC 7120 was grown in 100 mL Erlenmeyer flasks containing 25 mL of BG11_N_ medium containing 17.6 mM sodium nitrate [82] for 3 days on a shaker with continuous shaking at the rate of 63 rpm and at a light intensity of 18 µmol photons m^−2^s^−1^ and at 25 °C. Afterwards, cells were washed 3 times with nitrogen-free medium (BG11_0_) [82] to eliminate nitrogen traces. Then, cyanobacterium was grown in BG11_0_ medium for 48 h, in order to adduce nitrogen starvation and induce heterocyst formation, in two experimental versions: (1) the control samples were grown without the addition of aqueous BMAA solution and (2) the treated samples were grown with the addition of an aqueous solution of beta-N-methylamino-L-alanine (L-BMAA) (Cat no. B-107, Sigma-Aldrich, MO, USA) to a final concentration of 20 µM, as it was performed earlier in [13]. Later cells from both experimental versions were collected by centrifugation at 5000 rpm for 10 min at 4 °C and frozen at −80 °C until being used for proteomic analysis. The experiment was performed in 3 independent biological replicates.

The time of cell treatment with BMAA (48 h) was selected according to our previously published studies [13,14].

### 4.2. Trypsin Digestion in Solution

Cellular pellet was treated with lysozyme (0.3 mg/mL) (Sigma) for 60 min at 4 °C and resuspended in 100 μL 100 mM tris-HCl buffer, рН 8.0 with the addition of Protease inhibitor Mix (GE Healthcare), 0.1% sodium deoxycholate (DCNa) (Sigma) and 2.5 mM EDTA (Sigma). Cells were lysed by six cycles of 30 sec sonication (Cell Disruptor, Branson) and 5 min incubation at 4 °C. After that, dry urea and DCNawere added to the sample to final concentrations of 8 M and 1%, respectively. After incub ation for 20 min, the sample was centrifuged at 14,000 rpm for 10 min at 4 °C to remove intact cells. The supernatant was selected, and protein concentration was estimated using Bradford Protein Assay Kit (BioRad, Hercules, CA, USA). Protein cysteine bonds were reduced in the supernatant by the addition of 5 mM Tris (2-carboxyethyl) phosphine hydrochloride (TCEP) (Sigma, Saint Lois, MO, USA) for 60 min at 37 °C and, subsequently, alkylated with 30 mM iodoacetamide (BioRad) at room temperature in the dark for 30 min. The step in which TCEP was added was repeated. Then, the sample was diluted 6-fold with 50 mM Tris-HCl, pH 8.0 with 0.01% DCNa. Trypsin (Trypsin Gold, Mass Spectrometry Grade, Promega) was added in 1/50 w/w trypsin/protein ratio and incubated at 37 °C overnight. To stop trypsinolysis and degrade the acid-labile DCNa, trifluoroacetic acid (TFA) (Sigma) was added to a final concentration of 0.5% v/v (the pH should be less than 2.0), incubated at 37 °C for 45 min and the samples were centrifugated at 14,000× *g* for 10 min to remove the DCNa. Peptide extract was desalted using a Discovery DSC-18 Tube (Supelco) according to the manufacturer’s protocol. Peptides were eluted with 1 mL 75% acetonitrile (Sigma) with 0.1% TFA, dried in a SpeedVac (Labconco, Kansas City, MO, USA and resuspended in 3% acetonitrile with 0.1% TFA to a final concentration of 5 μg/μL.

### 4.3. LC-MS/MS Analysis

The analysis was performed on a Triple TOF 5600+ mass spectrometer with a NanoSpray III ion source (AB Sciex, Framingham, MA, USA) coupled with a NanoLC Ultra 2D+ nano-HPLC system (Eksigent, now part of Sciex, Framingham, MA, USA) as we have described [83]. The HPLC system was set up in trap-elute mode. The buffer A and the sample loading buffer consist of a mixture of 98.9% water, 1% methanol, 0.1% formic acid (v/v). Buffer B included 99.9% acetonitrile and 0.1% formic acid (v/v). Samples were loaded on a Chrom XP C18 trap column (3.6μm, 120 Å, 350 μm × 0.5 mm; Eksigent, Dublin, CA, USA ) at a flow rate of 3 μL/min for 10 min and eluted through a 3C18-CL-120 separation column (3 μm, 120 Å, 75 μm × 150 mm; Eksigent) at a flow rate of 300 nl/min. The gradient was performed from 5% to 40% buffer B in 90 min followed by 10 min at 95% buffer B and 20 min of reequilibration with 5% buffer B. To wash the system and to prevent carryover, two blank 45-min runs consisting of 5 to 8 min waves (5% B, 95%, 95%, 5%) were performed between the different samples.

The information-dependent mass-spectrometer experiment included one survey MS1 scan followed by 50 dependent MS2 scans. MS1 acquisition parameters were set up as follows: the mass range for MS2 analysis was 300–1250 m/z, and the signal accumulation time was 250 ms. Ions for MS2 analysis were selected on the basis of intensity with a threshold of 200 counts per second and a charge state from 2 to 5. MS2 acquisition parameters were as follows: the resolution of the quadrupole was set to UNIT (0.7 Da), the measurement mass range was 200–1800 m/z, and the signal accumulation time was 50 ms for each parent ion. Collision-activated dissociation was performed with nitrogen gas and the collision energy ranged from 25 to 55 V within the signal accumulation time of 50 ms. Analyzed parent ions were sent to the dynamic exclusion list for 15 s in order to get an MS2 spectra at the chromatographic peak apex. 

β-Galactosidase tryptic solution (20 fmol) was run with a 15-min gradient (5% to 25% buffer B) every two samples and between sample sets to calibrate the mass spectrometer and to control the overall system performance, stability, and reproducibility. 

### 4.4. Protein Identification by LC-MS/MS Data Analysis

For protein identification and semi-quantitative spectral counting, all LC-MS/MS data were searched against the National Center for Biotechnology Information (NCBI) GenBank protein sequence database for *Nostoc* sp. PCC 7120 also containing common contaminant proteins. Identification of proteins was performed with ProteinPilot (version 4.5, Sciex, ABSciex, Forster, CA, USA) in an identification mode with the following parameters: Cys alkylation by iodoacetamide, trypsin digestion, TripleTOF 5600 instrument, false discovery rate (FDR) analysis, and thorough ID search with a detected protein threshold of 95.0%. Protein identification was considered significant if the estimated local false discovery rate was 1% or lower, and at least 2 different peptides were identified for the protein with a confidence score above 95%. Spectral counting was performed with in-house script under emPAI [84] protocol with only tryptic peptides with local FDR ≤ 1% taken into account. 

Quantitative analysis was performed with MaxQuant against the same database. The settings used were as follows: a standard label-free analysis; fixed cysteine carbamidomethylation (which is allowed for use in quantitation); no variable modifications; default settings for Sciex Q-TOF instrument for MS and MS/MS spectra processing; tryptic digest with KP/RP cleavage prohibited and with 0 missed sites allowed; label-free quantification with minimum 2 label-free quantification (LFQ) ratios; normalization performed and missing peaks requantified; minimum peptide length 7, maximum peptide mass 4600 Da, only unique peptides used for quantification. The PSM and protein FDR threshold was set to 5%, and at least 1 unique peptide was required for the protein group. Statistical significance of observed differences in each case was assessed with Welch’s 2-sided t-test with Benjamini–Yekutieli adjustment for multiple comparisons with *p*-value thresholds of 0.05 and 0.1.

### 4.5. Pathway Analysis Based on LC-MS/MS Data

The significantly altered proteins obtained from LC-MS/MS data analysis were subjected to analysis using the UniProt Knowledgebase (https://web.expasy.org/docs/userman.html#what_is_sprot) and the Kyoto Encyclopedia of Genes and Genomes (KEGG) pathways database (https://www.genome.jp/kegg/pathway.html). 

Protein–protein interactions were analyzed by STRING (Protein–Protein Interaction Networks Functional Enrichment Analysis; https://string-db.org). Gene coexpression data for *Nostoc* (*Anabaena*) sp. PCC 7120 were obtained from ALCOdbCyano (http://alcodb.jp/cyano/). The coexpression data in this database were calculated using 116 microarray data items downloaded from the KEGG EXPRESSION Database (https://www.genome.jp/kegg/expression/). Sequence information and gene annotations were retrieved from GenomeNet Database Resources (https://www.genome.jp/).

NtcA-regulated genes were found with CollecTF database (a database of transcription factor binding sites (TFBS) in the Bacteria domain) (http://www.collectf.org/browse/home/) [85].

## Figures and Tables

**Table 1:** Beta-N-methylamino-L-alanine (BMAA) effect on protein profile of *Nostoc* during nitrogen starvation *.

No.	Pathway	Number of Proteins Affected by BMAA	Total Amount	
			Up Shifted	Down Shifted
1	Nitrogen metabolism	3	1	2
2	Heterocyst formation	2	1	1
3	CO_2_ fixation and CO_2_-concentrating mechanism	2	0	2
4	Carbohydrate metabolism, Glycolisis and gluconeogenesis	6	0	6
5	Photosynthesis	9	2	7
6	Amino acids metabolism	8	3	5
7	Signalling, Stress response, GTP-binding proteins and proteases	8	6	2
8	Chaperones	3	3	0
9	Nucleotide metabolism, purine and pyrimidine	4	2	2
10	DNA repair	4	4	0
11	Transcription	3	2	1
12	Ribosomal proteins	5	2	3
13	Translation	4	2	2
14	Secondary metabolites	4	0	4
15	ABC-transporters and transporters	3	1	2
16	Hypothetical proteins	35	18	17
	Total	103	47	56

* The number of upshifted and downshifted proteins is identified according to label-free quantification (LFQ) ratio of BMAA treated sample/Control sample.

**Table 2:** BMAA action on selected identified proteins based on the statistical significance of the observed differences in protein profile of *Nostoc* during nitrogen starvation.

No.	Protein	Gene	Function	Fold Regulation *	*p*-Value
				LFQ Ratio BMAA Treated Sample/Control Sample	
**Nitrogen metabolism (3 proteins)**					
1	nrtA	*alr0608*	nitrate-binding protein	1.76	0.002
2	glnB|P-II	*all2319*	nitrogen regulatory protein	0.55	0.09
3	nifD	*all1454*	molybdenum-iron protein subunit alpha in nitrogenase	0.54	0.047
**Heterocyst formation (two DNA-binding proteins)**					
4	Apb2	*all1939*	transcription regulation of hepA and hepC genes	0.63	0.08
5	Abp1	*all1940*	transcription regulation of hepA and hepC genes	1.49	0.078
**Nucleotides metabolism, purine and pyrimidine (4 proteins)**					
6	EC:2.7.4.6 nucleoside diphosphate kinase	*alr3402*	transferring phosphorus-containing groups	0.75	0.004
7	EC:2.1.2.2 phosphoribosylglycinamide formyltransferase-2	*alr1299*	purine metabolism	2.14	0.05
8	EC:2.1.2.3 3.5.4.10 | purH; bifunctional purine biosynthesis protein	*all3093*	purine metabolism	0.71	0.08
9	EC:1.1.1.205 inosine 5-monophosphate dehydrogenase	*alr0051*	purine metabolism	1.75	0.00013
**Secondary metabolites (4 proteins)**					
10	EC:2.2.1.7 1-deoxy-xylulose 5-phosphate synthase	*alr0599*	Thiamine metabolism; Terpenoid backbone biosynthesis	found only in control sample	0.00001
11	EC:2.8.1.10 | thiG thiazole synthase	*all3519*	Thiamine biosynthesis protein	found only in control sample	0.02
12	EC:1.17.7.1 1.17.7.3 | ispG 4-hydroxy-3-methylbut-2-en-1-yl diphosphate synthase	*all2501*	Terpenoid backbone biosynthesis	0.55	0.0012
13	EC:1.3.1.83 1.3.1.111 | chlP geranylgeranyl hydrogenase	*alr0128*	Porphyrin and chlorophyll metabolism; Terpenoid backbone biosynthesis	0.49	0.01
**Photosynthesis (9 proteins)**					
14	psaA photosystem I P700 chlorophyll a apoprotein A1	*alr5154*	photosystem I	0.38	0.01
15	petC cytochrome b6-f complex iron-sulfur subunit	*all2453*	cytochrome b6-f	1.75	0.024
16	psbW photosystem II 13kDa protein	*all0801*	photosystem II	found only in BMAA treated sample	0.04
17	petE plastocyanin	*all0258*	plastocyanin	0.58	0.06
18	cpcA phycocyanin alpha chain	*alr0529*	phycocyanin	0.56	0.075
19	apcD allophycocyanin B alpha chain	*all3653*	allophycocyanin	0.82	0.076
20	EC:4.2.1.24 delta-aminolevulinic acid dehydratase	*alr4380*	Porphyrin and chlorophyll metabolism	0.51	0.08
21	psaB photosystem I P700 chlorophyll a apoprotein A2	*alr5155*	photosystem I	0.26	0.08
22	psaF photosystem I subunit III	*all0109*	photosystem I	0.61	0.04
**ABC-transporters and transporters (3 proteins)**					
23	branched-chain amino acid transport system substrate-binding protein	*alr2535*	ABC transporters; Quorum sensing	0.59	0.00003
24	secY | preprotein translocase subunit	*all4197*	Quorum sensing	found only in control sample	0.03
25	peptide/nickel transport system substrate-binding protein	*alr0140*	periplasmic oligopeptide-binding ABC transporter Quorum sensing	1.61	0.038
**Amino acids metabolism (8 protein)**					
26	EC 4.3.1.19 threonine dehydratase	*alr4232*	Glycine, serine and threonine metabolism; Valine, leucine and isoleucine biosynthesis	found only in control sample	0.0008
27	EC 2.6.1.52 phosphoserine aminotransferase	*all1683*	Glycine, serine and threonine metabolism; Cysteine and methionine metabolism	0.62	0.01
28	EC 2.5.1.47 cysteine synthase A	*alr4416*	Cysteine and methionine metabolism	0.69	0.03
29	EC 2.3.3.13 leuA 2-isopropylmalate synthase	*alr4840*	Valine, leucine and isoleucine biosynthesis; Pyruvate metabolism	0.55	0.06
30	ureC urease subunit alpha	*alr3670*	Arginine biosynthesis; Purine metabolism	0.82	0.08
31	EC:2.6.1.16 | NodM glutamine--fructose-6-phosphate aminotransferase	*alr3464*	Alanine, aspartate and glutamate metabolism	2.20	0.002
32	EC:1.2.1.16 1.2.1.79 1.2.1.20 succinate-semialdehyde dehydrogenase / glutarate-semialdehyde dehydrogenase	*all3556*	Alanine, aspartate and glutamate metabolism; Lysine degradation	1.64	0.022
33	thrA homoserine dehydrogenase	*all4120*	Glycine, serine and threonine metabolism	1.73	0.05
**Chaperones (3 proteins)**					
34	dnaK molecular chaperone DnaK	*alr2990*	Folding, sorting and degradation	found only in BMAA treated sample	0.002
35	dnaK molecular chaperone DnaK	*alr1742*	Folding, sorting and degradation	1.23	0.09
36	groES co-chaperonin GroES	*alr3661*	Chaperones and folding catalysts	1.54	0.08
**Signalling, Stress response, GTP-binding proteins and proteases (8 proteins)**					
37	TypA	*all4140*	GTP-binding protein Ribosome –binding Stress responce	2.04	0.059
38	peroxiredoxin	*alr4641*	signaling and cellular processes; acting on a peroxide as acceptor	2.02	0.0015
39	EC:1.15.1.1 superoxide dismutase, Fe-Mn family	*all0070*	Acting on superoxide as acceptor	found only in BMAA treated sample	0.0028
40	EC:1.8.1.7 gor; glutathione reductase (NADPH)	*all4968*	Glutathione metabolism	1.45	0.047
41	EC:1.8.1.9 thioredoxin reductase	*all0737*	Selenocompound metabolism	2.22	0.07
42	AhpC/TSA family protein	*alr4404*	This family includes peroxiredoxin proteins	0.39	0.035
43	cyclic-di-GMP-binding protein	*all4662*	signaling and cellular processes	0.73	0.05
44	EC:3.4.24. | ftsH cell division protease	*all4936*	cell division protease FtsH	1.89	0.015
**Translation (4 proteins)**					
45	GTP-binding protein LepA	*all2508*	elongation factor	found only in control sample	0.05
46	EC:6.1.1.17 gltX | glutamyl-tRNA synthetase	*all3205*	Aminoacyl-tRNA biosynthesis; Porphyrin and chlorophyll metabolism	found only in control sample	0.005
47	ribosome recycling factor (rrf)	*alr1208*	Translation factors	1.37	0.086
48	EC:6.1.1.6 lysS lysyl-tRNA synthetase	*all4071*	Aminoacyl-tRNA biosynthesis	1.28	0.096
**Ribosomal proteins, (5 proteins)**					
49	rplX | 50S ribosomal protein L24	*asl4204*	genetic information processing	2.56	0.014
50	rpsJ | 30S ribosomal protein S10	*all4336*	genetic information processing	0.068	0.059
51	rpsF | 30S ribosomal protein S6	*all4802*	genetic information processing	1.27	0.06
52	rps7 | 30S ribosomal protein S7	*all4339*	genetic information processing	0.74	0.06
53	rplL | 50S ribosomal protein L7/L12	*alr5303*	genetic information processing	0.67	0.094
**Transcription (3 proteins)**					
54	EC:2.7.7.6 rpoB DNA-directed RNA polymerase subunit beta	*alr1594*	Transcription	1.52	0.05
56	EC:2.7.7.6 rpoC1 DNA-directed RNA polymerase subunit beta	*alr1595*	Transcription machinery	2.13	0.08
55	sigA | RNA polymerase sigma factor RpoD	*all5263*	Transcription; Transcription regulation; DNA-binding	0.39	0.0046
**DNA metabolism (4 proteins)**					
57	EC:5.6.2.2 DNA gyrase subunit A	*all0860*	DNA replication DNA repair and recombination	1.92	0.02
58	recA recombinase A	*all3272*	DNA repair and recombination	3.03	0.047
59	EC:5.6.2.2 gyrB DNA gyrase subunit B	*all5265*	Enzymes altering DNA conformation	1.56	0.08
60	nucA sugar-non-specific nuclease	*all7362;* *alr8011*	genetic information processing	2.63	0.08
**CO_2_ fixation and CO_2_-concentrating mechanism (two proteins)**					
61	rbcL EC:4.1.1.39 ribulose bisophosphate carboxylase	*alr1524*	Carbon metabolism	0.67	0.0008
62	ccmM carbon dioxide concentrating mechanism protein	*all0865*	Carbon metabolism	0.64	0.02
**Carbohydrate metabolism, Glycolisis and gluconeogenesis (6 proteins)**					
63	EC:2.7.2.3 phosphoglycerate kinase (pgk)	*all4131*	Glycolysis Gluconeogenesis	0.78	0.0008
64	nucleotide sugar epimerase	*all3509*	Sugar metabolism	0.59	0.07
65	EC:3.1.3.11 fructose-1,6-bisphosphatase I	*all4021*	Glycolysis Gluconeogenesis	0.76	0.006
66	EC:1.1.1.44 1.1.1.343 6-phosphogluconate dehydrogenase	*alr5275*	Pentose phosphate pathway, Glutathione metabolism	0.68	0.05
67	NADH-dependent butanol dehydrogenase	*alr4566*	Propanoate metabolism	0.73	0.05
68	Phosphoglucomutase	*all5089*	Glycogenolysis and Gluconeogenesis	0.77	0.057

* Fold regulation stands for BMAA/control ratio as it was done in [17], i.e., fold changes between BMAA-treated and control samples are shown (*p* < 0.1). Additional information about 164 identified BMAA-regulated proteins, whose fold regulation was detected with 0.1 < *p* < 1 (not significant) and with, N.A. i.e. not available value, because only in one or two sample replications the peptide was detected, thereby *p*-value could not be calculated, is presented in Tables S4 and S5, as a Supplementary.

**Table 3:** Protein–protein interactions * between several strongly BMAA-affected proteins and ribosomal proteins, according to STRING (https://string-db.org/).

Ribosomal Proteins	Identified BMAA Regulated Proteins Involved in Translation				
	gltX Absent	lepA Absent	SecY Absent	rpsJ (S10) Strongly Downshifted	rplX (L24) Upshifted
**L2**	+	+		+	
L3		+		+	
L4					+
L5	+	+			+
**L6**	+	+	+		
L14	+	+			+
L15					+
L16				+	+
L18				+	+
L20		+			
L22				+	+
L29					+
S3				+	
**S5**		+	+	+	
S7				+	
S8	+		+		+
S9		+			
S11			+		
S12	+				
**S13**	+		+	+	
S17					+
S19				+	

* Protein–protein interaction is marked as “+”.

## Supplementary Materials

The following are available online at https://www.mdpi.com/2072-6651/12/5/310/s1, Figure S1: The impact of BMAA on nitrogen metabolism in nitrogen starving cells of *Nostoc* 7120., Figure S2: BMAA addition down regulates key enzymes of carbon fixation in nitrogen starving cells of *Nostoc* 7120, Figure S3: Protein network of ccmM and its protein partners, Figure S4: Glycolysis/Gluconeogenesis pathway, Figure S5: Pentose phosphate pathway, Figure S6: BMAA completely inhibits two key thiamine enzymes in nitrogen-starving cells of *Nostoc*7120, Figure S7: Photosynthesis, Figure S8: Porphyrin and Chlorophyll metabolism, Figure S9: Alanine, aspartate and glutamate metabolism, Figure S10: The protein network of glnA, glnB (PII), NodM and the other protein partners, according to STRING (https://string-db.org), Figure S11: BMAA disturbs amino acid metabolism in nitrogen-starving cells of *Nostoc* 7120, Table S1: Original proteomics data, Table S2: Results of BMAA effects on Hypothetical protein profile of *Nostoc* sp. PCC 7120 during nitrogen starvation, Table S3: Gene coexpression data for identified hypothetical proteins in proteome of nitrogen starving *Nostoc*sp. PCC 7120 under BMAA treatment according to ALCOdbCyano, Table S4: BMAA impact on protein profile of *Nostoc* during nitrogen starvation, Table S5: BMAA effect on protein profile of *Nostoc* during nitrogen starvation (LFQ ratio of BMAA treated sample/Control sample (0.1 < *p* < 1 (not significant)).
