# Supplementary material for: The First Proteomic Study of Nostoc sp. PCC 7120 Exposed to Cyanotoxin BMAA under Nitrogen Starvation
Source: Toxins (Basel). 2020 May 9;12(5):310. doi: 10.3390/toxins12050310 (PMC7290344; doi:10.3390/toxins12050310)
Supplement: Supplementary file 1 [file toxins-12-00310-s001.zip › toxins-787861-supple-author revised/toxins-787861-supplementary_corrected_ more small corrections.pdf]

Supplementary Materials: The First Proteomic Study of *Nostoc* sp. PCC 7120 Exposed to Cyanotoxin BMAA under Nitrogen Starvation

Olga A. Koksharova, Ivan O. Butenko, Olga V. Pobeguts, Nina A. Safronova, and Vadim M. Govorun

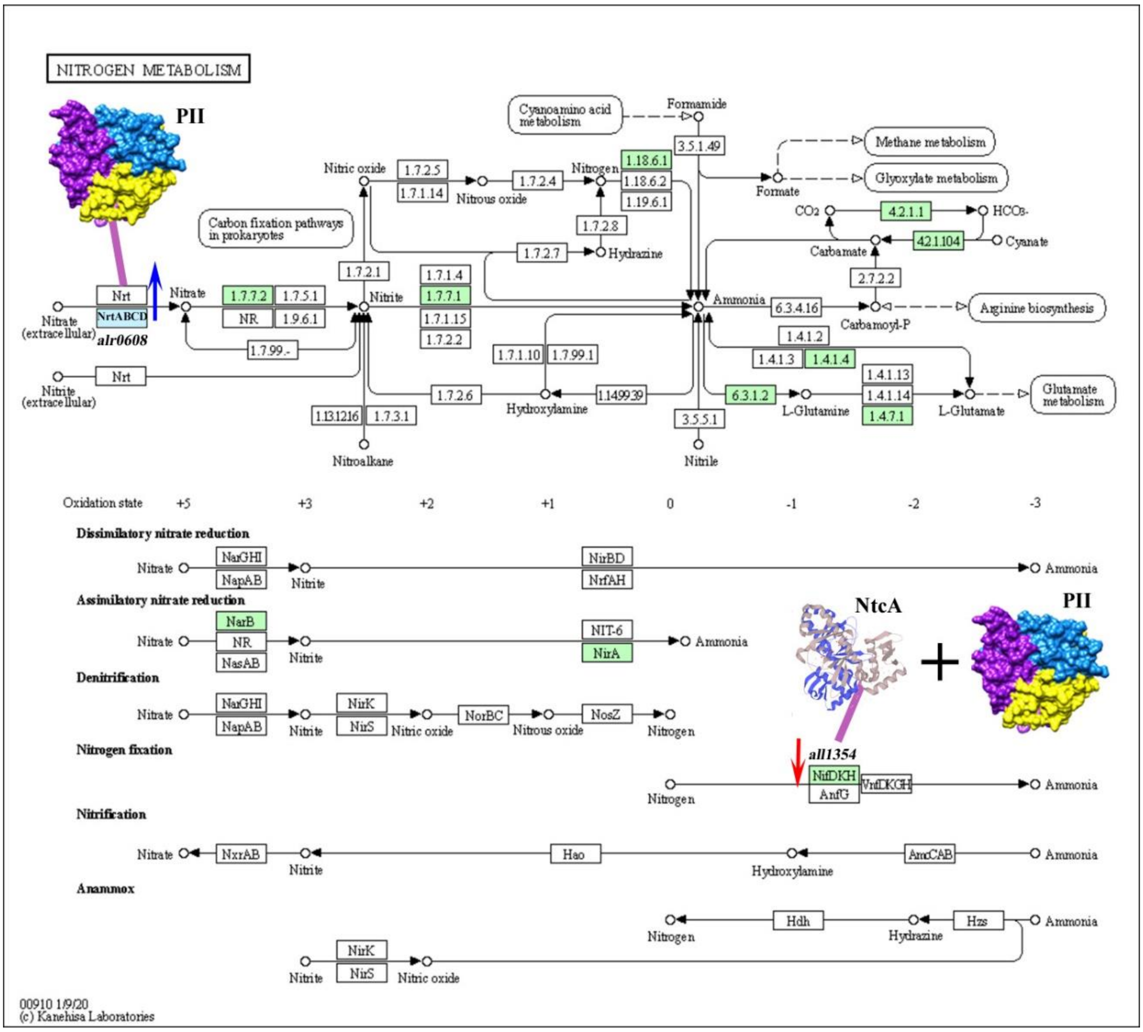

Figure S1. The impact of BMAA on nitrogen metabolism in nitrogen starving cells of *Nostoc* 7120. The Nitrogen metabolism KEGG pathway map is presented (https://www.genome.jp/kegg-bin/show_pathway?ana00910+alr0608) with modifications. Nitrate transport nitrate-binding protein (NrtA, *alr0608*) is upshifted and nifD protein (*all1354*) is downshifted at BMAA presence. Possible PII regulation is indicated.

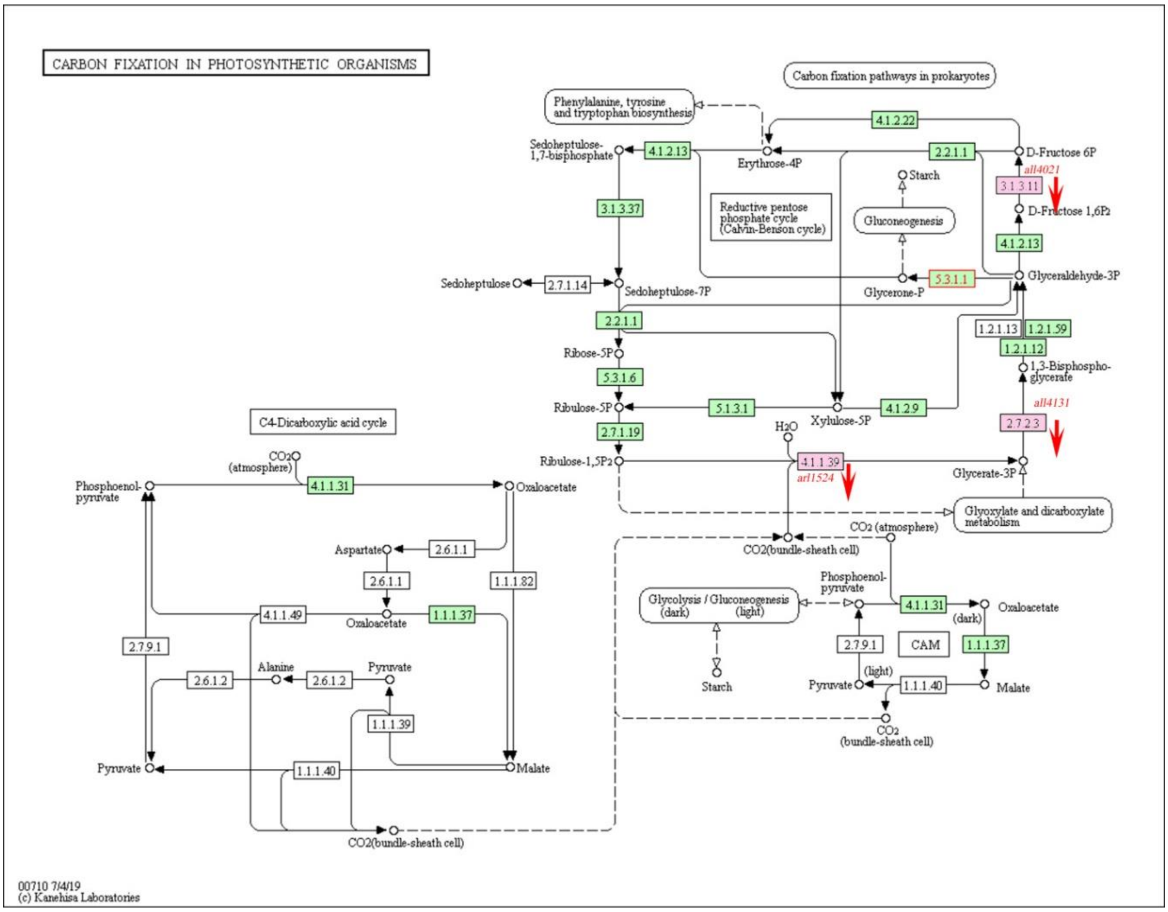

Figure S2. BMAA addition down regulates key enzymes of carbon fixation in nitrogen starving cells of *Nostoc* 7120. Three down-shifted proteins are indicated on this map (https://www.genome.jp/kegg-bin/show_pathway?ana00710+alr1524). Among them are rbcL (large subunit of ribulose bisphosphate carboxylase (EC:4.1.1.39, gene *alr1524*), phosphoglycerate kinase (EC:2.7.2.3, pgk, *all4131*) and fructose-1,6-bisphosphatase I (EC:3.1.3.11, *all4021*).

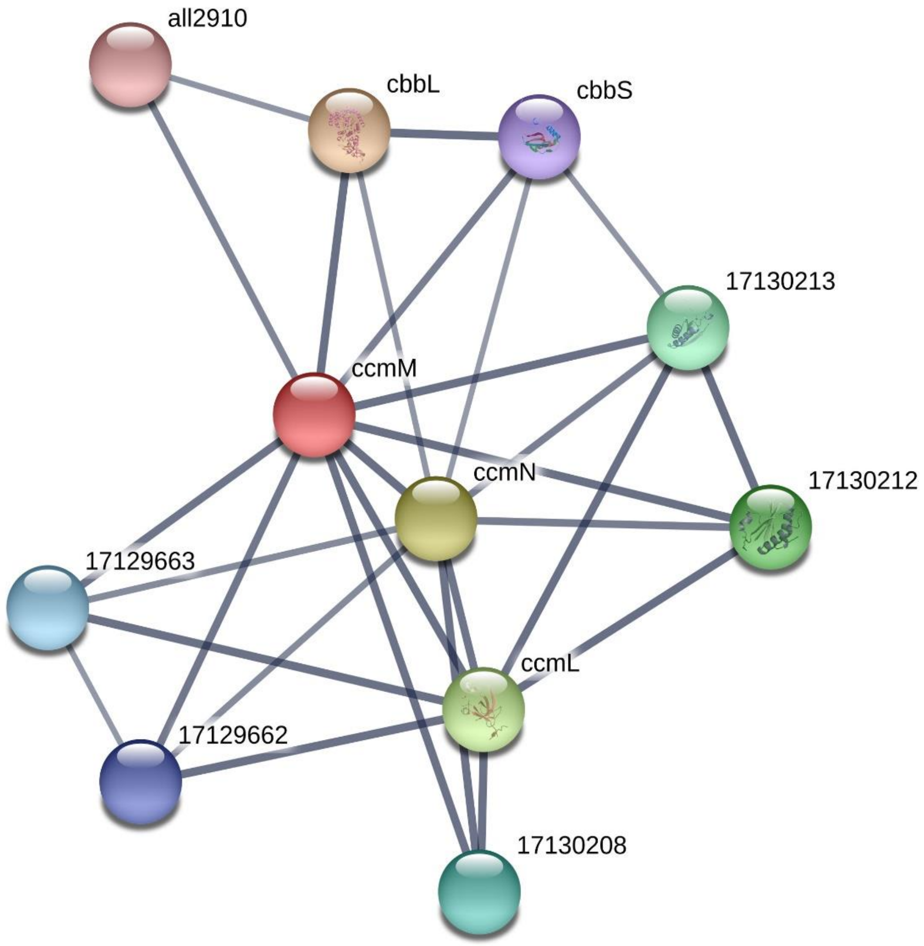

Figure S3. Protein network of ccmM and its protein partners. According to STRING (https://string-db.org), the ccmM protein interacts with the Ribulose bisphosphate carboxylase large chain (rbcL). Both proteins are found down regulated in nitrogen starving cyanobacterial cells under BMAA treatment. On the scheme are indicated cbbL, Ribulose bisphosphate carboxylase large chain; cbbS, Ribulose bisphosphate carboxylase small chain; all2910, Carbonic anhydrase; ccmM, ccmN and ccmL are carbon dioxide concentrating mechanism proteins; 17130208, 17130212, 17130213, 17129662, 17129663 are carbon dioxide concentrating mechanism proteins ccmK .

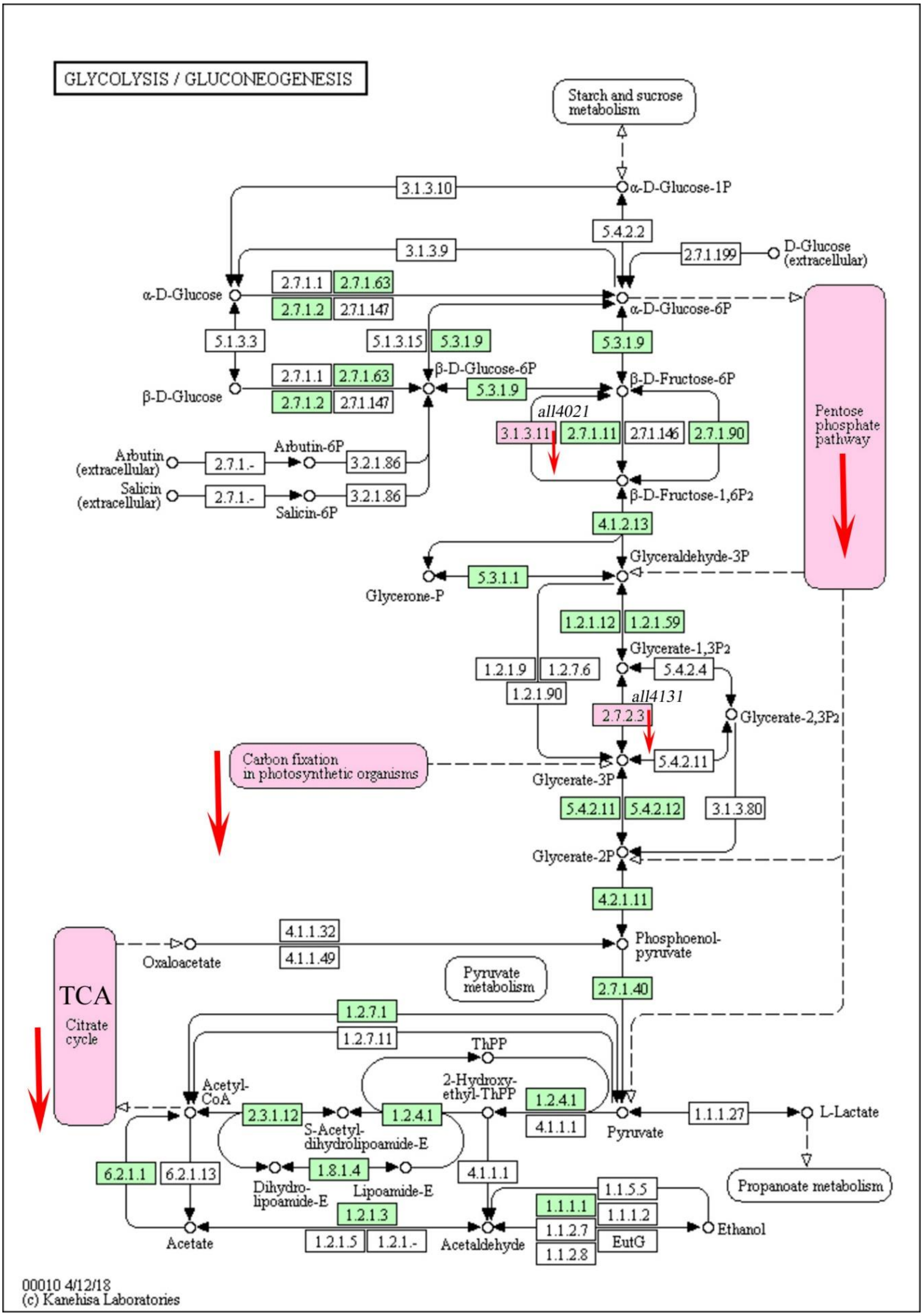

Figure S4. The Glycolysis/Gluconeogenesis pathway. Two identified downshifted proteins are marked on this scheme: fructose-1,6-bisphosphatase I (EC:3.1.3.11, *all4021*) and phosphoglycerate kinase (EC:2.7.2.3, pgk, *all4131*). Others down regulated pathways are indicated as well. (https://www.genome.jp/kegg-bin/show_pathway?ana00010+all4021).

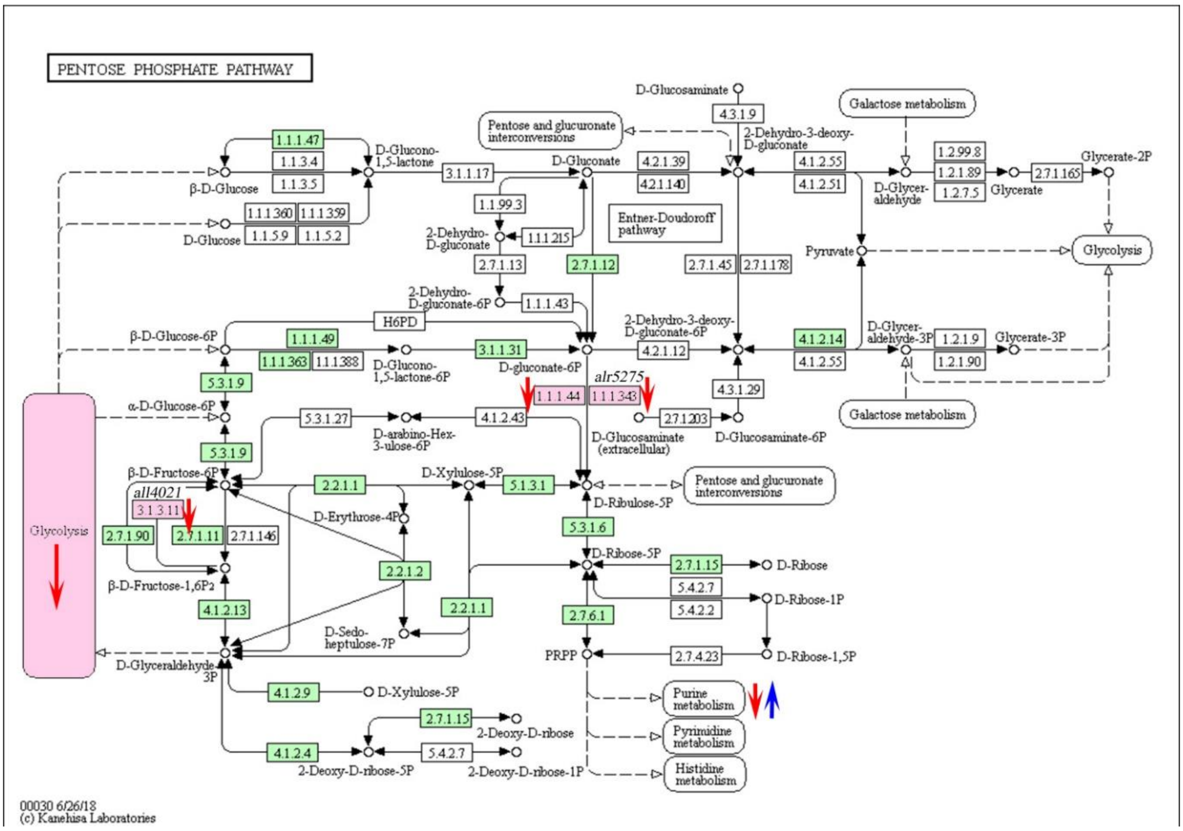

Figure S5. Pentose phosphate pathway. Two enzymes of pentose phosphate pathway are down-regulated at BMAA presence in *Nostoc* cells during nitrogen starvation. They are the 6-phosphogluconate dehydrogenase (EC:1.1.1.44 1.1.1.343, *alr5275*) and the fructose-1,6-bisphosphatase I (EC:3.1.3.11, *all4021*). (https://www.genome.jp/kegg-bin/show_pathway?ana00030+alr5275).

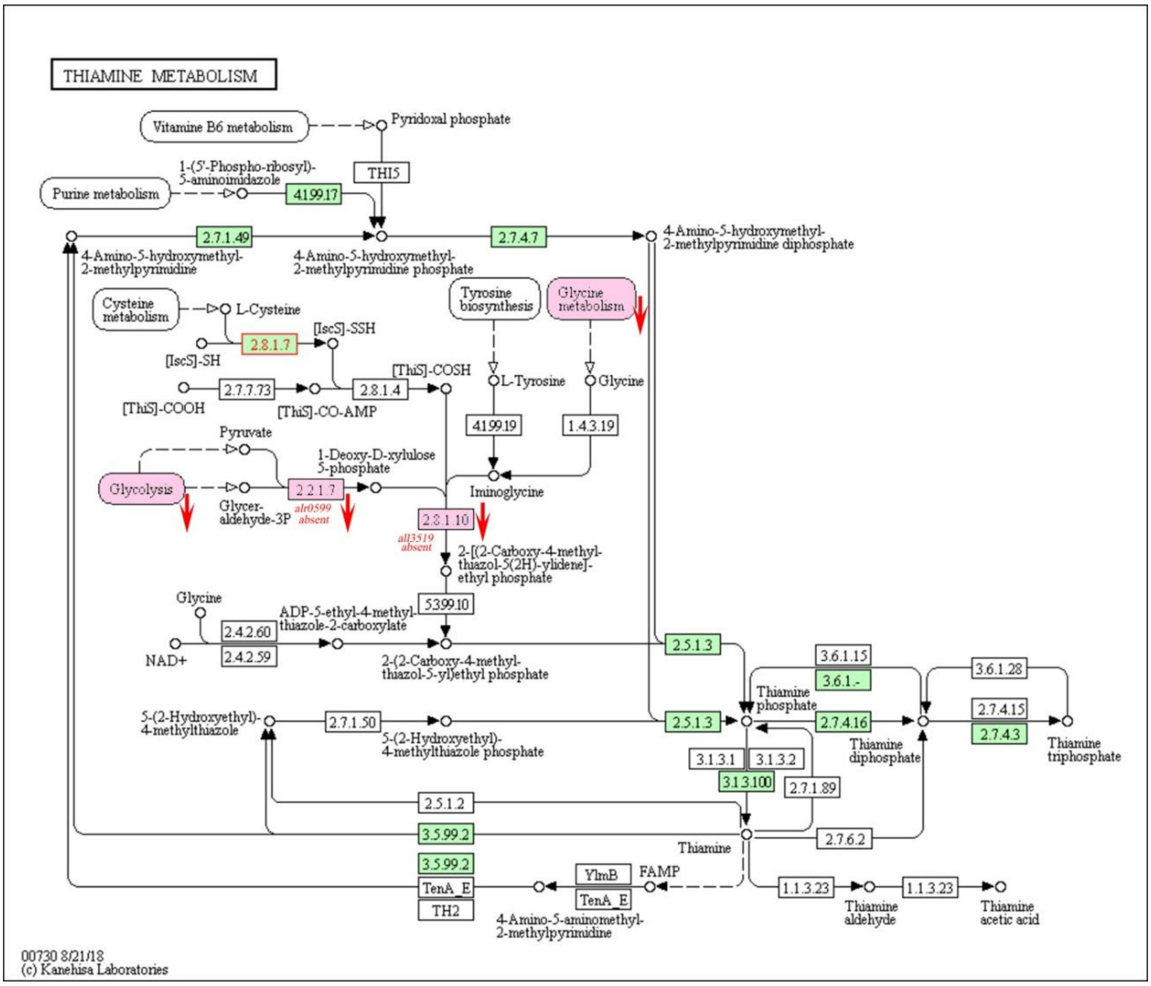

Figure S6. BMAA completely inhibits two key thiamine enzymes in nitrogen-starving cells of *Nostoc* 7120: 1-deoxy-xylulose 5-phosphate synthase (EC:2.2.1.7, *alr0599*) and thiazole synthase (thiG, EC:2.8.1.10, *all3519*), which participate in the same pathway as thiamine biosynthesis. They are absent in the BMAA-treated *Nostoc* cell samples. (https://www.genome.jp/kegg-bin/show_pathway?ana00730+alr0599).

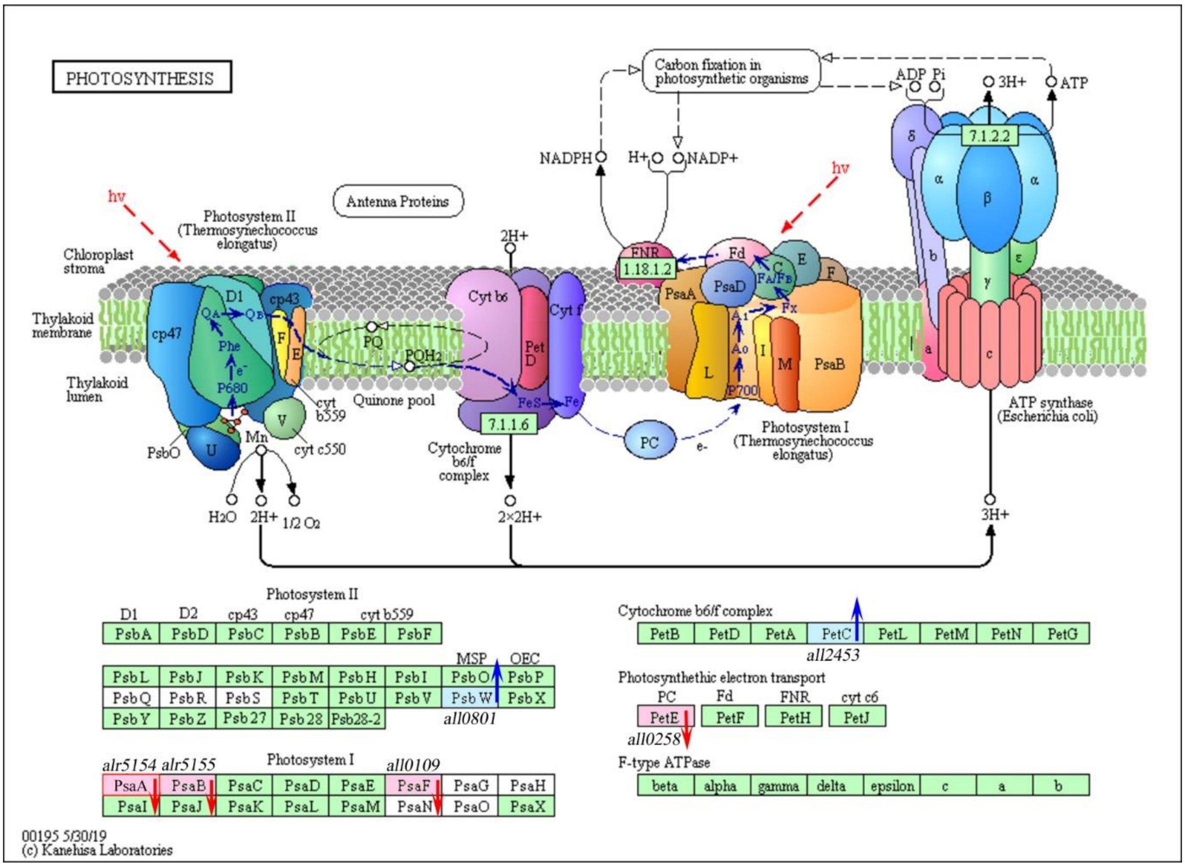

Figure S7. Photosynthesis. KEGG scheme of photosynthesis presents the main components of cyanobacterial photosynthetic apparatus. The main components of PSI (PsaA, PsaB and PsaF) and plactocyanin PetE are downshifted, while cytochrome b6-f complex iron-sulfur subunit PetC and small protein PsbW of PS2 are upregulated in BMAA treated cells. (https://www.genome.jp/kegg-bin/show_pathway?ana00195+alr5154).

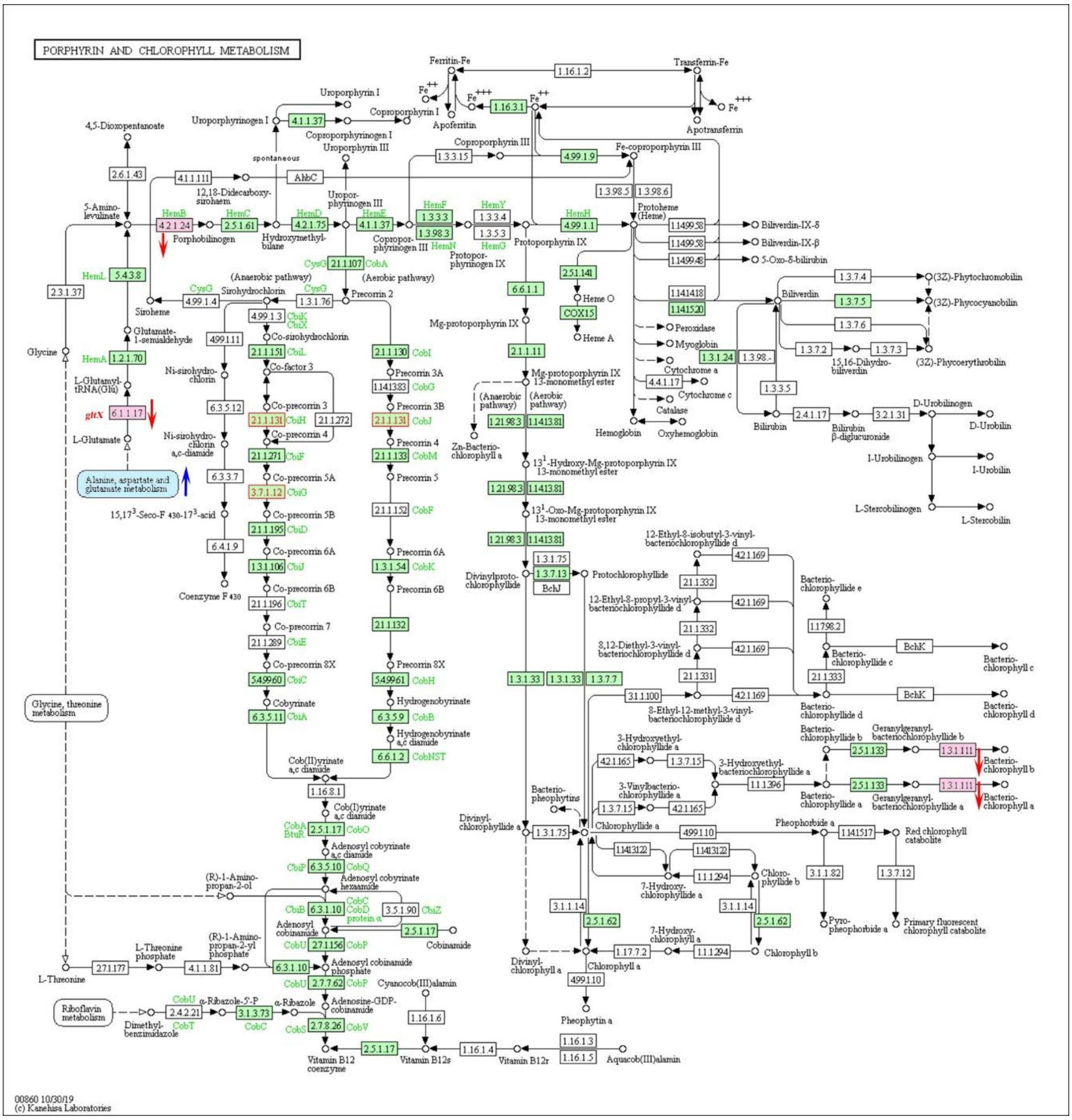

Figure S8. Porphyrin and Chlorophyll metabolism. Several enzymes involved in chlorophyll metabolism are down regulated in starving cyanobacterial cells at BMAA presence. They are delta-aminolevulinic acid dehydratase (EC:4.2.1.24, HemB, *alr4380*), glutamyl-tRNAsynthetase (EC:6.1.1.17, gltX, *all3205*), geranylgeranyl hydrogenase (EC:1.3.1.83 1.3.1.111, chlP, *alr0128*). (https://www.genome.jp/kegg-bin/show_pathway?ana00860+all3205).

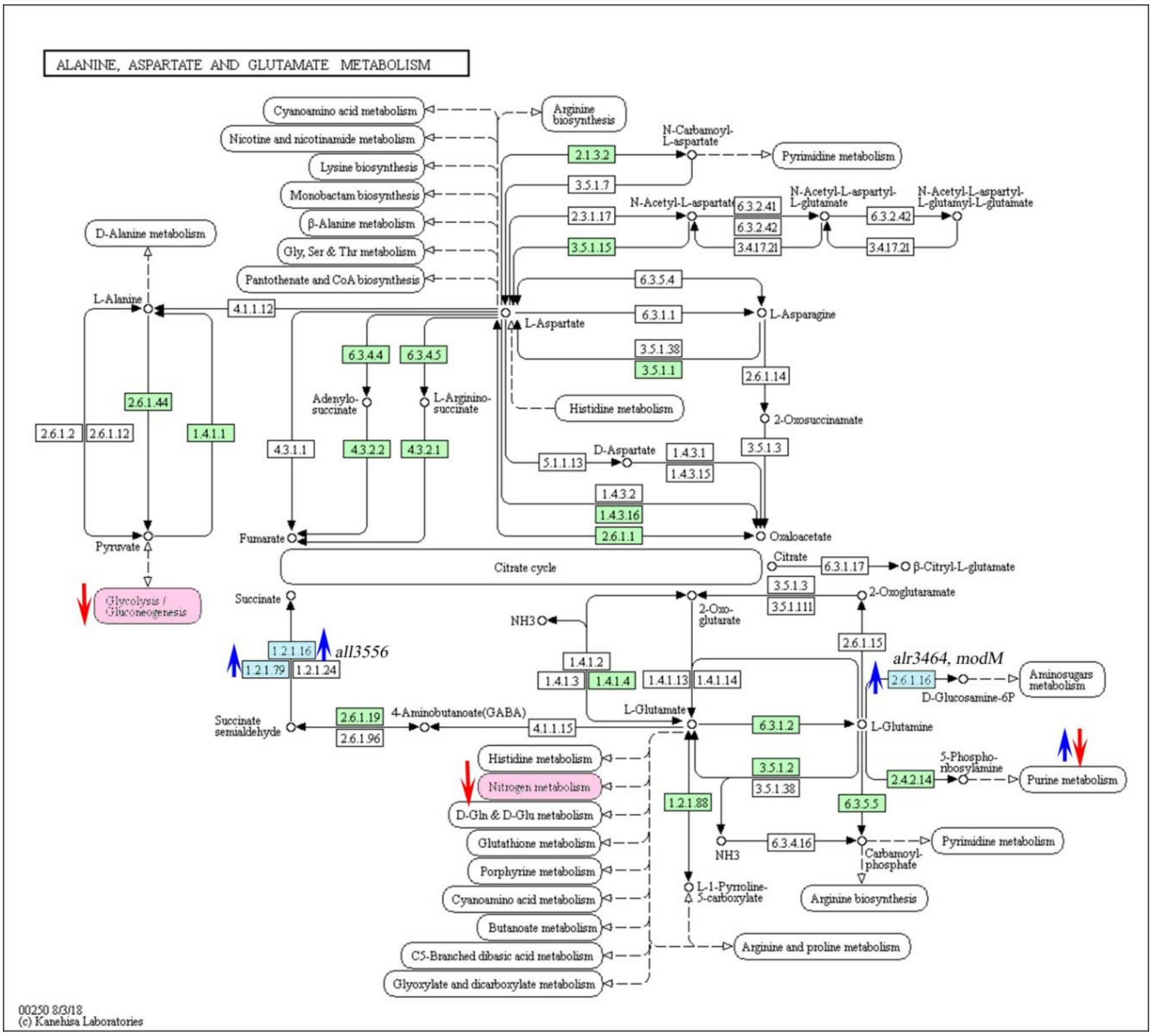

Figure S9. Alanine, aspartate and glutamate metabolism. Two enzymes, glucosamine-fructose-6-phosphate aminotransferase (NodM, EC:2.6.1.16, *alr3464*) and succinate-semialdehyde dehydrogenase/ glutarate-semialdehyde dehydrogenase (EC: EC:1.2.1.16, 1.2.1.79, *all3556*) are up-regulated at BMAA presence in nitrogen-starving cells of *Nostoc.* (https://www.genome.jp/kegg-bin/show_pathway?ana00250+alr3464).

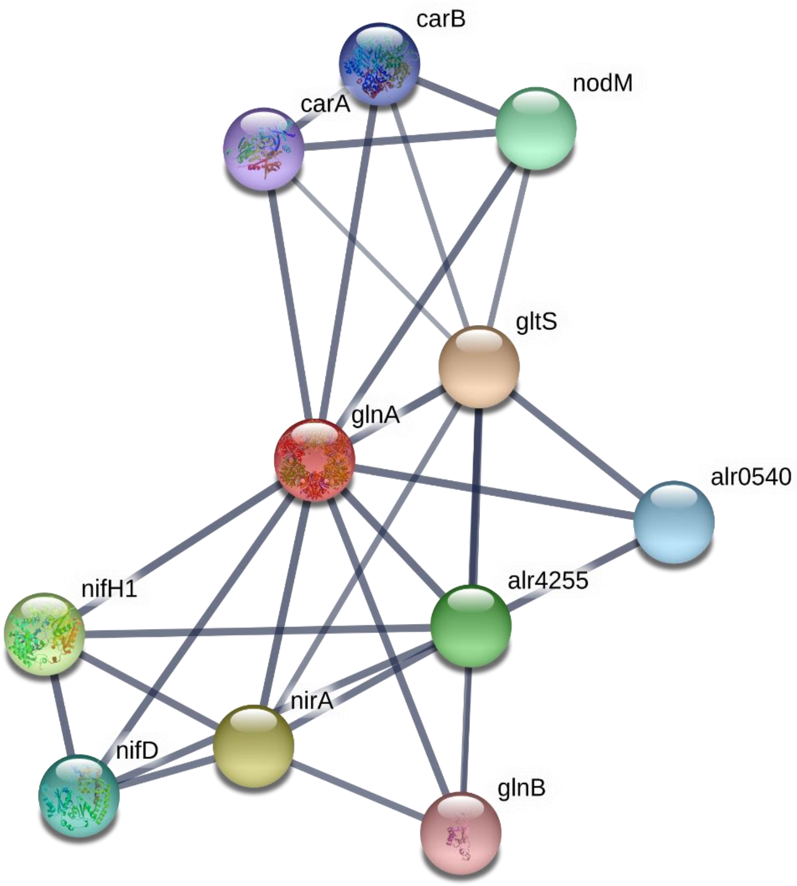

Figure S10. The protein network of glnA, glnB (PII), NodM and the other protein partners, according to STRING (https://string-db.org). Among them are Carbamoyl-phosphate synthase small chain (carA), Carbamoyl-phosphate synthase large chain (carB), Ferredoxin-glutamate synthase (gltS), nitrite reductase (nirA), Nitrogenase iron protein 1 (nifH1), Nitrogenase molybdenum-iron protein alpha chain (nifD), Glutamate dehydrogenase (alr4255), 1-pyrroline-5 carboxylate dehydrogenase (alr0540).

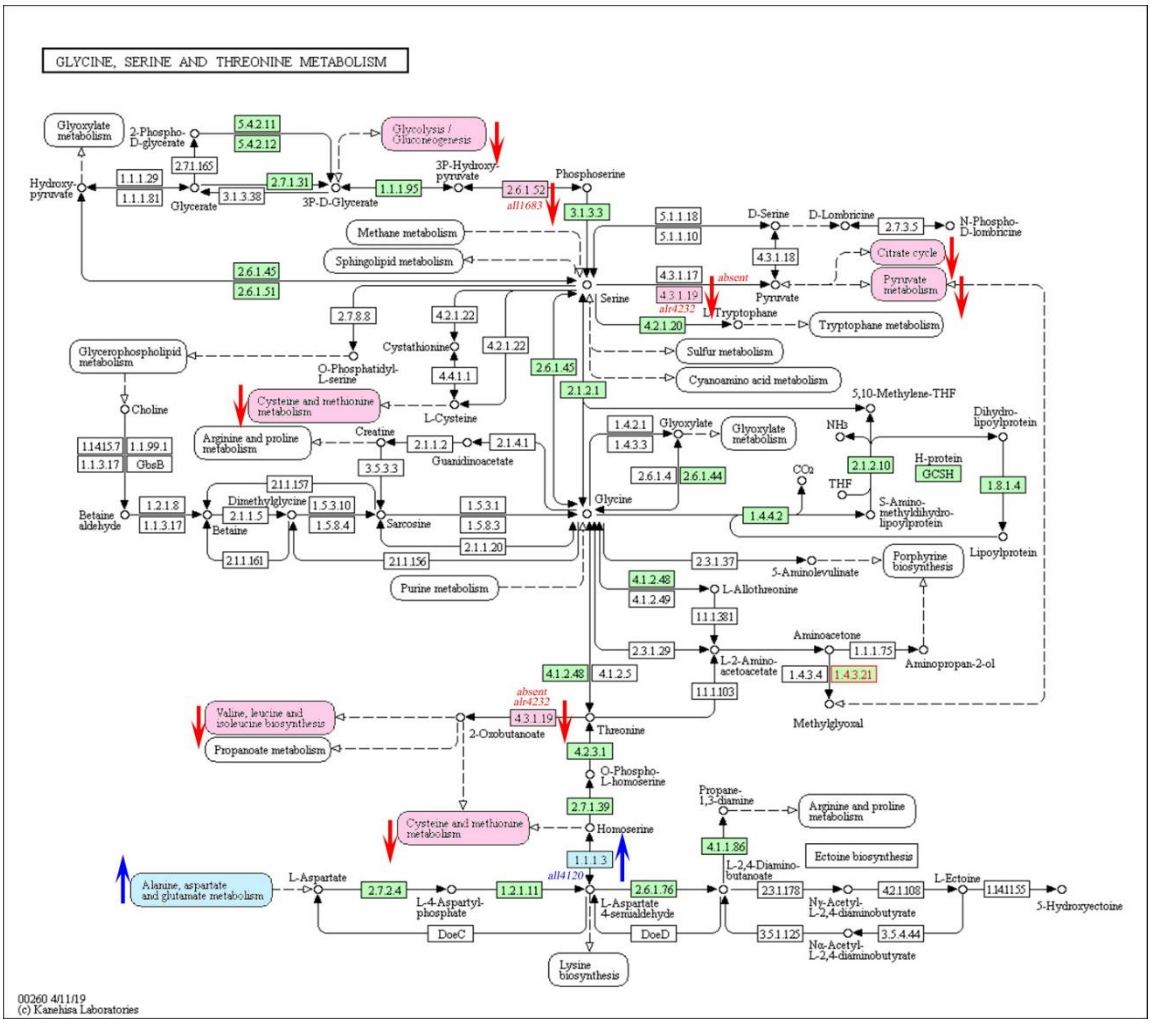

Figure S11. BMAA disturbs amino acid metabolism in nitrogen-starving cells of *Nostoc* 7120. The Kyoto Encyclopedia of Genes and Genomes (KEGG) pathway map of glycine, serine, and threonine (https://www.genome.jp/kegg-bin/show_pathway?ana00260+all1683) represents enzymes and biochemical processes affected by BMAA in nitrogen-depleted cells of cyanobacterium *Nostoc*. Arrows show downregulation (red) and upregulation (blue).

Table S2. Results of BMAA effects on Hypothetical protein profile of *Nostoc* sp. PCC 7120 during nitrogen starvation (fold regulationshows BMAA/control as done in [17], i.e., fold changes between BMAA-treated and control samples are shown (p< 0.1)). LFQ, label-free, "Control" indicates that the protein was present only in the control samples, but not in BMAA treated samples.

№	Gene	LFQ ratio BMAA treated/Control	*p*-value
		**Hypothetical proteins (35 proteins)**	
1	*alr1346*	3.41	0.0003
2	*all1872*	1.85	0.0089
3	*alr4505*	6.67	0.0092
4	*all1411*	4.55	0.0102
5	*alr3106*	1.54	0.0169
6	*all3526*	2.7	0.0173
7	*asl4547*	1.72	0.0188
8	*all1865*	1.79	0.0202
9	*alr0652*	2.7	0.0215
10	*alr2889*	1.32	0.0451
11	*all4916*	1.43	0.0262
12	*alr0740*	1.92	0.0366
13	*all1272*	1.96	0.0455

14	*all3984*	1.82	0.0462
15	*asr3294*	1.59	0.0735
16	*alr4514*	1.52	0.0947
17	*all1861*	1.64	0.0787
18	*all4916*	1.41	0.0262
19	*asl4369*	found only in control sample	0.0005
20	*all1361*	0.39	n/d
21	*alr3101*	0.43	0.0453
22	*alr7074*	0.53	0.0245
23	*alr2440*	0.59	0.0530
24	*all2080*	0.63	0.0345
25	*all0065*	0.64	0.0024
26	*all1237*	0.5	0.0097
27	*all4018*	0.59	0.0207
28	*all3054*	0.83	0.0333
29	*all2375*	0.71	0.0718
30	*all1975*	0.46	0.0018
31	*alr3297*	0.67	0.0923
32	*alr4787*	0.53	0.0209
33	*all2050*	0.83	0.0527
34	*all4662*	0.73	0.0512
35	*alr2440*	0.59	0.0530

Table S3. Gene coexpression data for identified hypothetical proteins in proteome of nitrogen starving *Nostoc*sp. PCC 7120 under BMAA treatment according to ALCOdbCyano (http://alcodb.jp/cyano/) are shown. The coexpressed genes encoding proteins identified in this study are marked by green.

**Up-shifted hypothetical proteins**			
**№**	**Hypothetical gene**	**Pair similar regulated proteins (this study)**	**List of coexpressed genes from the ALCOdbCyano Database**
1	*alr1346*	*alr1346*–*all4936*	DnaK-type molecular chaperone; DnaK (*alr1742*)<br>Putative modulator of DNA gyrase; TldD (*all5219*)<br>Small heat shock protein (*alr0286*)<br>Group 2 sigma 70-type sigma factor E (*alr3800*)<br>Cell division protein FtsH (*all4936*)<br>Heat shock protein GrpE (*alr2445*)
2	*all1411*	*all1411*–*all0860*	33kD chaperonin, heat shock protein HSP33 (*all1588*)<br>DNA-binding protein, starvation-inducible (*all1173*)<br>DNA gyrase A subunit (*all0860*)
3	*alr4505*	ND*	DnaJ protein (*alr2991*)
4	*all3526*	ND	Cobalamin biosynthesis protein D (all2847)
5	*alr0652* (mrp protein homolog)	ND	50S ribosomal protein L13 (*all4188*)<br>Chaperonin GroEL (*alr1896*)<br>30S ribosomal protein S9 (*all4187*)
6	*all1272* (probable glycogen phosphorylase)	ND	UDP-N-acetylmuramoylalanyl-D-glutamate--2,6-diamino pimelate ligase (*all1663*)
**Down-shifted hypothetical proteins**			
1	*all2375* (similar to bacterioferritin comigratory protein)	*all2375*–*all2319*<br>*all2375*–*all2080*	Nitrogen regulatory protein P-II (*all2319*)<br>Hypothetical protein (*all2080*)
2	*all2080*	*all2080*–*all2375*	Similar to bacterioferritin comigratory protein (*all2375*)

		all2080–*all2319*	(this study)<br>Manganese-stabilzing protein precursor (*all3854*)<br>Nitrogen regulatory protein P-II (*all2319*)<br>Carbon dioxide concentrating mechanism protein CcmK (*alr0318*)
3	*alr2440*	*alr2440*–*alr4404*	AhpC/TSA family protein (*alr4404*)<br>Heterocyst differentiation protein HetR (*alr2339*)<br>Allophycocyanin beta subunit; ApcB (*alr0022*)<br>Carbon dioxide concentrating mechanism protein CcmK (*alr0317*)<br>Phycobilisome core-membrane linker protein ApcE (*alr0020*)
4	*all1361* (similar to cytochrome P450)	*all1361*–*all0865*	Carbon dioxide concentrating mechanism protein CcmM (*all0865*)<br>Carbon dioxide concentrating mechanism protein CcmK (*all0867*)
5	*alr3101*	ND	ABC transporter ATP-binding protein (*alr2486*)
6	*alr3297*	ND	Plastoquinol--plastocyanin reductase, cytochrome b6; PetB (*alr3421*)<br>30S ribosomal protein S20 (*asr1592*)<br>Dihydrolipoamide dehydrogenase (*alr4745*)<br>Heterocyst differentiation related protein PatN (*alr4812*)<br>Glutamine ABC transporter, glutamine-binding protein GlnH (*alr3429*)
7	*all4018* (putative OxPPCycle protein OpcA)	ND	Glucose 6-phosphate dehydrogenase (*all4019*)<br>ABC nitrate transport permease protein NrtB (*all3335*)
8	*alr4787*	ND	Putative acetyl transferase

ND*—coexpressed proteins from ALCOdbCyanolist are not detected together in this study

Table S4. BMAA impact on protein profile of *Nostoc* during nitrogen starvation*.

№	Pathway	Total amount **		Total amount***	
		**Up shifted**	**Down shifted**	**Up shifted**	**Down shifted**
**1**	Nitrogen metabolism	1	2	0	1
2	Heterocyst formation	1	1	0	2
3	CO_2 fixation and CO_2-concentrating mechanism	0	2	1	0
4	Carbohydrate metabolism, Glycolisis and gluconeogenesis	0	6	8	11
5	Photosynthesis	2	7	5	13
6	Amino acids metabolism	3	5	3	10
7	Signalling, Stress response, GTP-binding proteins and proteases	6	2	5	9
8	Chaperones	3	0	0	0
9	Nucleotide metabolism, purine and pyrimidine	2	2	2	1
10	DNA repair and metabolism	4	0	2	1
11	Transcription	2	1	4	2
12	Ribosomal proteins	2	3	2	7
13	Translation	2	2	1	4
14	Secondary metabolites	0	4	2	0
15	ABC-transporters and transporters	1	2	1	9
16	Hypothetical proteins	18	17	30	28

Total	47	56	66	98

*The number of upshifted and downshifted proteins is identified according to label-free quantification (LFQ) ratio of BMAA treated sample/Control sample ($p< 0.1$)**(data from Table 1). Additionally, data for BMAA-regulated proteins, detected with $0.1 < p < 1$ *** are shown (more detail data is presented in Table S5).

Table S5. BMAA effect on protein profile of *Nostoc* during nitrogen starvation (LFQ ratio of BMAA treated sample/Control sample ($0.1 < p < 1$ (not significant)). N.A. i.e. not available value, because only in one or two sample replications the peptide was detected, thereby *p*-value could not be calculated.

№	Protein	Gene	Function	Fold regulation LFQ ratio BMAA treated sample/control sample	*p*-value
1	magnesium chelatase subunit I [EC:6.6.1.1]	All0152	Porphyrin and chlorophyll metabolism	0.61	0.13
2	hypothetical	All0415	unknown	0.65	0.29
3	hypothetical	All0405	unknown	0.72	0.178
4	hypothetical	All0646	unknown	0.68	0.256
5	ribose 5-phosphate isomerase A [EC:5.3.1.6]	All0888	Pentose phosphate pathway	0.52	0.129
6	nitrogenase molybdenum-iron protein beta chain [EC:1.18.6.1]	All1440	Heterocyst functions and Nitrogen metabolism	0.36	0.184
7	K02588 nitrogenase iron protein NifH	All1455	Heterocyst functions and Nitrogen metabolism	0.15	0.351
8	glutathione-dependent peroxiredoxin [EC:1.11.1.27]	All1541	oxidative stress defence	1.45	0.566
9	K01928 UDP-N-acetylmuramoyl-L-alanyl-D-glutamate--2,6-dia minopimelate ligase [EC:6.3.2.13	All1663	Lysine biosynthesis	0.61	0.108
10	NAD(P)H-quinone oxidoreductase subunit M [EC:7.1.1.2]	All1732	Oxidative phosphorylation	0.67	0.166
11	protochlorophyllide reductase [EC:1.3.1.33]	All1743	Porphyrin and chlorophyll metabolism	0.74	0.201
12	hypothetical	All1751		0.68	0.196
13	similar to NADH dehydrogenase	All1864	NitroreductaseTM1586_NiRd ase	0.58	0.572
14	Unknown protein	All 2002	unknown	1.85	0.399
15	Unknown protein	All2086	unknown	0.74	0.152
16	FMN-dependent NADH-azoreductase [EC:1.7.1.17] acpD; acyl carrier protein phosphodiesterase	all2105	Oxidoreductases Acting on other nitrogenous compounds as donors The enzyme catalyses the reductive cleavage of an azo bond in aromatic azo compounds to form the corresponding amines. Does not accept NADPH.	1.63	0.612
17	(GenBank) similar to chloroplast membrane-associated 30 kD protein	all2342	photosynthesis	1.52	0.560
18	thioredoxin 1	all2367	regulation	0.69	0.153
19	hypothetical protein	all2705	unknown	0.73	0.219
20	unknown protein	all3041	unknown	0.73	0.363
21	hypothetical protein	all3116	unknown	0.67	0.277
22	hypothetical protein	all3144	unknown	0.76	0.258

23	two-component hybrid sensor and regulator	all3764	Regulatory	1.46	0.233
24	uroporphyrinogen decarboxylase [EC:4.1.1.37] hemE	all3909	Porphyrin and chlorophyll metabolism	0,60	0,207
25	thiosulfate/3-mercaptopyruvate sulfurtransferase [EC:2.8.1.1 2.8.1.2]	all3918	Cysteine and methionine metabolism	0.37	0.178
26	unknown	all3941	unknown	0.58	0.213
27	pyruvate kinase [EC:2.7.1.40]	all4008	Glycolysis / Gluconeogenesis	0.78	0.259
28	polar amino acid transport system substrate-binding protein ǀ (GenBank) binding protein of ABC transporter component	all4130	signaling and cellular processes	0.48	0.199
29	rps5; 30S ribosomal protein S5	all4199	translation	1.55	0.276
30	rpl6; 50S ribosomal protein L6	all4201	translation	0.75	0.211
31	rps3; 30S ribosomal protein S3	all4209	translation	1.35	0.472
32	rpl22; 50S ribosomal protein L22	all4210	translation	0.77	0.334
33	peptide chain release factor 3	all4379	translation	0.56	0.148
34	phosphoadenosine phosphosulfate reductase [EC:1.8.4.8 1.8.4.10]	all4464	Sulfur metabolism	0,76	0.420
35	phosphoribosylformimino-5-aminoimidazole carboxamide ribotide isomerase [EC:5.3.1.16] ǀ	all4506	Histidine metabolism	0.74	0.271
36	fructose-bisphosphate aldolase, class II [EC:4.1.2.13]	all4563	Glycolysis / Gluconeogenesis	0.73	0.129
37	acetolactate synthase I/II/III large subunit [EC:2.2.1.6]	all4613	Valine, leucine and isoleucine biosynthesis	1.41	0.421
38	translation initiation factor IF-3	all4623	translation	0.63	0,382
39	hypothetical protein	all4664	unknown	0,75	0.271
40	hypothetical protein	all4894	unknown	1.71	0.190
41	histidyl-tRNA synthetase [EC:6.1.1.21]	all5012	translation	0.80	0.326
42	unknown protein	all5018	unknown	1.69	0.155
43	biopolymer transport protein ExbB	all5047	signaling and cellular processes	0.62	0.343
44	acetyl-CoA carboxylase biotin carboxyl carrier protein ǀ (GenBank) accB; biotin carboxyl carrier protein	all5057	Fatty acid biosynthesis	0.39	0.123
45	unknown protein	all7185	unknown	0.61	0.191
46	sugar-non-specific nuclease inhibitor NuiA homolog	all7262	NuiA homolog Nuclease inhibitor	0.62	0.119
47	hypothetical protein	all7372	unknown	0.54	0.445
48	unknown protein	all7598	unknown	0.37	0.157
49	unknown protein	all7607	unknown	0.34	0.376
50	thioredoxin 1 ǀ (GenBank) trxA; thioredoxin	alr0052	regulation	0.34	0.161
51	single-strand DNA-binding protein	alr0088	Replication and repair	1.64	0.131
52	pecC; phycoerythrocyanin-associated rod linker protein	alr0525	photosynthesis antenna proteins	1.51	0.193

53	cpcG3; phycobilisome rod-core linker protein	alr0536	Photosynthesis - antenna proteins	1.71	0.217
54	nrtC; nitrate transport ATP-binding protein	alr0610	Nitrogen metabolism	0.63	0.131
55	carbon dioxide concentrating mechanism protein ccmK	all0867	Carbon fixation	1.45	0.125
56	oligopeptidase A [EC:3.4.24.70]	alr0880	peptidase	0.78	0.616
57	hypothetical protein	alr0882	unknown	1.59	0.177
58	glucose-6-phosphate isomerase [EC:5.3.1.9]	alr1050	Glycolysis / Gluconeogenesis	1.45	0.212
59	uridylate kinase [EC:2.7.4.22]	alr1207	Pyrimidine metabolism	2.01	0.196
60	ATP-binding cassette, subfamily B, bacterial ABC transporter ATP-binding protein	alr1554	signaling and cellular processes	2.37	0.237
61	deaminated glutathione amidase [EC:3.5.1.128]	alr2001	is involved in clearing cells of the toxic compound deaminated glutathione	0.71	0.259
62	diaminopimelate epimerase [EC:5.1.1.7] |	alr2048	Lysine biosynthesis	0.70	0.364
63	phosphoribosylaminoimidazole-succinocarboxamide synthase [EC:6.3.2.6]	alr2268	Purine metabolism	0.62	0.310
64	chloroplastic outer envelope membrane protein homolog	alr2269	signaling and cellular processes	0.81	0.495
65	hypothetical protein	alr2309	unknown	1.49	0.162
66	superoxide dismutase, Fe-Mn family [EC:1.15.1.1] | (GenBank) sodB; iron superoxide dismutase	alr2938	Oxidative stress response	0.71	0.232
67	hypothetical	alr3199	unknwn	0.25	0.245
68	hypothetical protein	alr3277	unknown	1.62	0.225
69	magnesium-protoporphyrin IX monomethyl ester (oxidative) cyclase [EC:1.14.13.81]	alr3300	Porphyrin and chlorophyll metabolism	0.73	0.475
70	YidC/Oxa1 family membrane protein insertase	alr3415	Quorum sensing	0.77	0.115
71	phosphoribosylamine---glycine ligase [EC:6.3.4.13]	alr3510	Purine metabolism	1.48	0.246
72	hypothetical protein	alr3529	unknown	0.70	0.210
73	long-chain acyl-CoA synthetase [EC:6.2.1.3]	alr3602	Fatty acid biosynthesis	1.46	0.501
74	similar to endoglucanase	alr3608	Glucose polymer metabolism	0.75	0.199
75	hypothetical protein	alr3790	unknown	0.76	0.583
76	transcription termination/antitermination protein NusA	alr3829	transcription	0.74	0.345
77	iron complex transport system substrate-binding protein | (GenBank) ABC transporter iron binding protein	alr3938	signaling and cellular processes	0.76	0.454
78	NAD(P)H-quinone oxidoreductase subunit 5 [EC:7.1.1.2] ndhF	alr3956	Oxidative phosphorylation	0.68	0.329
79	similar to isovaleryl-CoA dehydrogenase	alr4058	oxidoreductase	1.51	0.577
80	demethylphylloquinone reductase [EC:1.6.5.12]	alr4094	Secondary metabolites Ubiquinone and other terpenoid-quinone	1.46	0.617

			biosynthesis		
81	S-adenosylmethionine synthetase [EC:2.5.1.6]	alr4124	Cysteine and methionine metabolism	1.48	0.217
82	general L-amino acid transport system substrate-binding protein | (GenBank) periplasmic amino acid-binding protein of amino acid ABC transporter	alr4164	Transport signaling and cellular processes	0.71	0.218
83	glutamate N-acetyltransferase / amino-acid N-acetyltransferase [EC:2.3.1.35 2.3.1.1] | (GenBank) argJ; ornithine acetyl transferase	alr4235	Arginine biosynthesis	0.77	0.398
84	malate dehydrogenase (oxaloacetate-decarboxylating) [EC:1.1.1.38]	alr4596	Pyruvate metabolism Arbon metabolism	0.66	0.171
85	preprotein translocase subunit SecA [EC:7.4.2.8] | (GenBank) secA; preprotein translocase SecA subunit	alr4851	Quorum sensing Protein export	0.71	0.146
86	LL-diaminopimelate aminotransferase [EC:2.6.1.83]	alr5103	Lysine biosynthesis	0.73	0.167
87	protein Tex | (GenBank) probable transcription accessory protein	alr5249	transcription	1.35	0.642
88	transcription termination/antitermination protein NusG | (GenBank) nusG; transcription antitermination protein	alr5299	transcription	1.98	0.123
89	large subunit ribosomal protein L11	alr5300	translation	0.76	0.676
90	Unknown protein	alr7208	unknown	1.58	0.530
91	Hypothetical protein	alr7502	unknown	3.69	0.246
92	Hypothetical protein	alr7524	unknown	0.73	0.285
93	glutaredoxin 3	asl3860	regulation	0.66	0.159
94	rbpD; RNA-binding protein	asl4022	regulation	1.50	0.258
95	hypothetical protein	asl4482	unknown	0.70	0.228
96	apcC; phycobilisome core linker protein Lc7.8	asr0023	Photosynthesis—antenna proteins	0.73	0.339
97	rod-capping linker polypeptide, phycocyanin-associated	asr0531	Photosynthesis - antenna proteins	0.65	0.225
98	hypothetical protein	asr1156	unknown	1.43	0.139
99	acyl carrier protein	asr3342	Biosynthesis of secondary metabolites	1.58	0.279
100	photosystem I subunit VII | (GenBank) psaC; photosystem I iron-sulfur protein	asr3463	photosynthesis	0.88	0.726
101	DNA-binding protein HU-beta	asr3935	transcription	0.77	0.131
102	uncharacterized protein	asr4959	unknown	1.56	0.158
103	uncharacterized protein	all0089	unknown	1.60	N.A.
104	UDP-N-acetylglucosamine 1-carboxyvinyltransferase [EC:2.5.1.7] |	all0174	Amino sugar and nucleotide sugar metabolism	0.69	N.A.
105	methionyl-tRNA synthetase [EC:6.1.1.10]	all0233	translation	0.42	N.A.
106	unknown protein	all0459	unknown	3.04	N.A.

107	1,4-alpha-glucan branching enzyme [EC:2.4.1.18	all0875	Carbon metaboilsm	1.94	N.A.
108	D-alanine-D-alanine ligase [EC:6.3.2.4]	all0980	D-Alanine metabolism	0.60	N.A.
109	putative chitobiose transport system substrate-binding protein periplasmic sugar-binding protein of ABC transporter	all1027	Transport, signaling and cellular processes	0.48	N.A.
110	uncharacterized protein ǀ (GenBank) btpA; biogenesis of thylakoid protein A	all1221	Function unknown	2.42	N.A.
111	hypothetical protein	all1355	unknown	0.78	N.A.
112	similar to cytochrome P450	all1361	heme binding, integral component of membrane	0.40	N.A.
113	two-component response regulator	all1736	regulatory	0.61	N.A.
114	hypothetical protein	all2108	unknown	0.57	N.A.
115	hypothetical protein	all2333	unknown	1.85	N.A.
116	unknown protein	all2425	unknown	1.84	N.A.
117	glyceraldehyde 3-phosphate dehydrogenase [EC:1.2.1.12] gap1	all2566	Carbon metabolism	0.49	N.A.
118	hypothetical protein	all2716	unknown	0.43	N.A.
119	S-(hydroxymethyl)glutathione dehydrogenase / alcohol dehydrogenase [EC:1.1.1.284 1.1.1.1]	all2810	Glycolysis / Gluconeogenesis	0.68	N.A.
120	ATP phosphoribosyltransferase regulatory subunit ǀ (GenBank) hisS; histidyl-tRNA synthetase	all2915	Histidine metabolism translation	0.63	N.A.
121	ABC transport protein, ATP-binding subunit	all3132	transport protein	0.59	N.A.
122	glucose-6-phosphate 1-dehydrogenase [EC:1.1.1.49 1.1.1.363] ǀ (GenBank) zwf; glucose 6-phosphate dehydrogenase	all4019	Carbon metabolism	2.04	N.A.
123	similar to carboxyl-terminal processing proteinase	all4090	Protein modification	2.95	N.A.
124	homoserine dehydrogenase [EC:1.1.1.3] ǀ thrA;	all4120	Glycine, serine and threonine metabolism	1.73	N.A.
125	large subunit ribosomal protein L13 ǀ (GenBank) rpl13	all4188	translation	0.65	N.A.
126	small subunit ribosomal protein S11 ǀ rps11; 30S	all4192	translation	0.73	N.A.
127	hypothetical protein	all4303	unknown	1.29	N.A.
128	aminomethyltransferase [EC:2.1.2.10] ǀ (GenBank) glycine cleavage system protein T	all4609	Glycine, serine and threonine metabolism	0.52	N.A.
129	hypothetical protein	all4749	unknown	1.40	N.A.
130	N-acetylmuramoyl-L-alanine amidase [EC:3.5.1.28] ǀ (GenBank) N-acetylmuramoyl-L-alanine amidase	all4999	Peptidoglycan biosynthesis and degradation proteins	1.34	N.A.
131	hypothetical protein	all5274	unknown	2.23	N.A.
132	unknown protein	all7065	unknown	1.32	N.A.

133	unknown protein	all7373	unknown	1.67	N.A.
134	citrate synthase [EC:2.3.3.1] (GenBank) gltA; citrate synthase	alr0222	Citrate cycle (TCA cycle)	2.16	N.A.
135	phosphate acyltransferase [EC:2.3.1.274] plsX; fatty acid/phospholipid synthesis	alr0238	Glycerolipid metabolism	0.65	N.A.
136	UDP-glucuronate decarboxylase [EC:4.1.1.35] | (GenBank) rfbB; dTDP-glucose 4-6-dehydratase	alr0657	Amino sugar and nucleotide sugar metabolism	0.65	N.A.
137	hypothetical protein	alr0871	unknown	5.06	N.A.
138	unknown protein	alr1144	unknown	0.56	N.A.
139	acetylglutamate kinase [EC:2.7.2.8] | (GenBank) argB; acetylglutamate kinase	alr1245	Arginine biosynthesis	2.93	N.A.
140	hypothetical protein	alr1537	unknown	2.25	N.A.
141	hypothetical protein	alr1613	unknown	1.58	N.A.
142	similar to zeta-carotene desaturase; unknown protein	alr1788	unknown	2.71	N.A.
143	unknown protein	alr1910	unknown	2.50	N.A.
144	unknown protein	alr2055	unknown	0.67	N.A.
145	two-component hybrid sensor and regulator	alr2428	regulatory	3.15	N.A.
146	unknown protein	alr2558	unknown	0.35	N.A.
147	unknown protein	alr2735	unknown	0.62	N.A.
148	DNA topoisomerase I [EC:5.6.2.1]	alr2780	DNA metabolism	1.64	N.A.
149	hypothetical protein	alr2927	unknown	2.35	N.A.
150	thioredoxin-dependent peroxiredoxin [EC:1.11.1.24]	alr3183	Acting on a peroxide as acceptor	0.71	N.A.
151	unknown protein	alr3301	unknown	2.35	N.A.
152	serine protease [EC:3.4.21.-]	alr3543	regulatory	0.52	N.A.
153	asparaginyl-tRNA synthetase [EC:6.1.1.22]	alr3658	translation	1.64	N.A.
154	hypothetical protein	alr3950	unknown	0.43	N.A.
155	hypothetical protein	alr4017	unknown	0.71	N.A.
156	rbpD; RNA-binding protein	alr4683	regulation	2.00	N.A.
157	acetyl-CoA carboxylase carboxyl transferase subunit alpha [EC:6.4.1.2 2.1.3.15] |	alr5285	Fatty acid biosynthesis	1.91	N.A.
158	unknown protein	alr7504	unknown	1.41	N.A.
159	unknown protein	alr7506	unknown	1.83	N.A.
160	photosystem I subunit IX | (GenBank) psaJ; photosystem I subunit IX	asl0108	Photosynthesis	0.71	N.A.
161	hypothetical protein	asl4862	unknown	1.58	N.A.
162	small subunit ribosomal protein S21 | (GenBank) rps21; 30S ribosomal protein S21	asr0742	translation	0.39	N.A.
163	small subunit ribosomal protein S16 | (GenBank) rps16; 30S	asr1953	translation	0.53	N.A.
164	CAB/ELIP/HLIP superfamily of protein	asr5262	light-harvesting-like protein 3	4.68	N.A.
